# Integrative Multi-PTM Proteomics Reveals Dynamic Global, Redox, Phosphorylation, and Acetylation Regulation in Cytokine-Treated Pancreatic Beta Cells

**DOI:** 10.1016/j.mcpro.2024.100881

**Published:** 2024-11-15

**Authors:** Austin Gluth, Xiaolu Li, Marina A. Gritsenko, Matthew J. Gaffrey, Doo Nam Kim, Priscila M. Lalli, Rosalie K. Chu, Nicholas J. Day, Tyler J. Sagendorf, Matthew E. Monroe, Song Feng, Tao Liu, Bin Yang, Wei-Jun Qian, Tong Zhang

**Affiliations:** 1 Biological Sciences Division, Pacific Northwest National Laboratory, Richland, Washington, USA; 2 Department of Biological Systems Engineering, Washington State University, Richland, Washington, USA

**Keywords:** multi-PTM, SP3, cysteine thiol oxidation, phosphorylation, acetylation

## Abstract

Studying regulation of protein function at a systems level necessitates an understanding of the interplay among diverse posttranslational modifications (PTMs). A variety of proteomics sample processing workflows are currently used to study specific PTMs but rarely characterize multiple types of PTMs from the same sample inputs. Method incompatibilities and laborious sample preparation steps complicate large-scale physiological investigations and can lead to variations in results. The single-pot, solid-phase–enhanced sample preparation (SP3) method for sample cleanup is compatible with different lysis buffers and amenable to automation, making it attractive for high-throughput multi-PTM profiling. Herein, we describe an integrative SP3 workflow for multiplexed quantification of protein abundance, cysteine thiol oxidation, phosphorylation, and acetylation. The broad applicability of this approach is demonstrated using cell and tissue samples, and its utility for studying interacting regulatory networks is highlighted in a time-course experiment of cytokine-treated β-cells. We observed a swift response in the global regulation of protein abundances consistent with rapid activation of JAK-STAT and NF-κB signaling pathways. Regulators of these pathways as well as proteins involved in their target processes displayed multi-PTM dynamics indicative of complex cellular response stages: acute, adaptation, and chronic (prolonged stress). PARP14, a negative regulator of JAK-STAT, had multiple colocalized PTMs that may be involved in intraprotein regulatory crosstalk. Our workflow provides a high-throughput platform that can profile multi-PTMomes from the same sample set, which is valuable in unraveling the functional roles of PTMs and their co-regulation.

## Highlights

• SP3 is compatible with proteomics analysis of cysteine thiol oxidation.
• An automated SP3-based workflow facilitates high-throughput multi-PTM profiling.
• Cytokines induces time-dependent regulation of protein abundance and PTMs.
• Interplay of thiol oxidation, phosphorylation, and acetylation on selected proteins.

## Graphical Abstract

**Figure:**
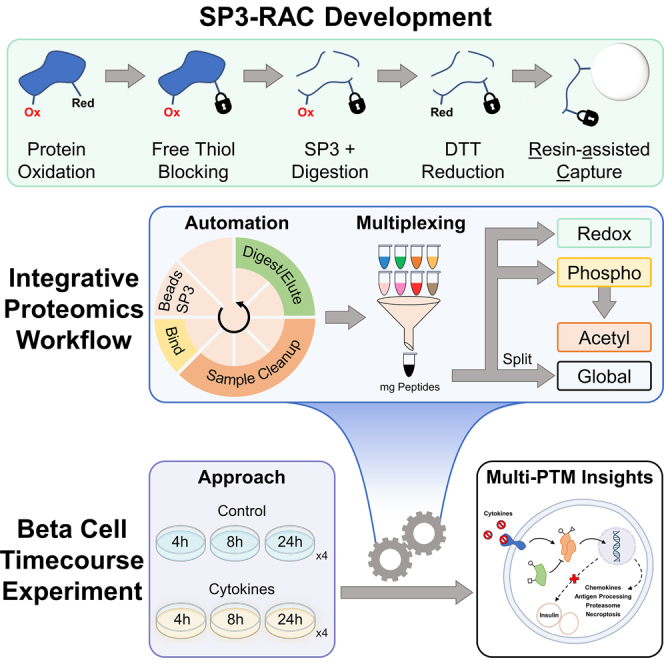

## In Brief

An automated universal sample processing method was developed for multi-PTM profiling, including protein thiol oxidation, phosphorylation, and acetylation. The method was used to unravel the functional roles of PTMs and their coregulation in cytokine signaling in β-cells.

The overall objective of proteomics is to detail the protein complement of a biological system functioning under specific conditions. Because protein structure, location, stability, and molecular interactions beget function, protein identification and quantification provide only a marginal view of these systems. Posttranslational modifications (PTMs) regulate protein function and communicate extracellular and intracellular phenomena such as nutrient availability and redox states (1, 2). A multitude of different PTMs work in concert to modulate physiological processes, which vastly expands the complexity of proteomes. Conversely, dysregulation of multiple types of PTMs has been observed in many human diseases including diabetes, cancer, and Alzheimer’s (3, 4, 5, 6, 7, 8, 9). Recent reports of the interplay (*i.e.*, crosstalk) among phosphorylation and those with redox PTMs on cysteine thiols provide excellent examples of how protein function is controlled by multiple types of PTMs (10). Phosphorylation is the most widely studied PTM due to its significance in cell signaling. Redox PTMs on cysteine thiols are being increasingly recognized for their roles in reactive oxygen species–mediated signaling (1, 3, 11, 12). At the molecular level, redox PTMs such as disulfide bonds and S-glutathionylation can inhibit or boost phosphorylation events in intertwined signal transduction pathways (13, 14, 15). Ultimately, multiple types of PTMs can coexist on the same protein, and their combinatorial actions provide complex regulatory mechanisms for protein function (16, 17, 18).

A landscape view of multiple types of PTMs is absent in most bottom-up proteomics studies, thereby impeding efforts to decode PTM interplay and advance theories about how pathophysiology emerges from dysregulation (9, 16). Nevertheless, various groups have made important contributions to this space in the past 2 decades (19, 20, 21, 22, 23, 24, 25, 26, 27, 28, 29, 30, 31, 32, 33). Strategies such as serial enrichment, in which peptides in the flowthrough of one enrichment step are used for subsequent enrichments, have increased the amount of PTM information that can be attained from a single experiment (30, 34). Overall, few of these studies investigate the interplay between cysteine thiol oxidation and other types of PTMs: Su *et al*. processed different sets of samples to enrich cysteine-containing peptides and phosphopeptides separately, while Huang *et al*. do not exclusively enrich cysteine-containing peptides (13, 24, 25). Moreover, none of the aforementioned studies harness automated sample preparation. An integrative multi-PTM proteomics approach would allow one to seamlessly integrate automated cleanup, digestion, and multiplexing steps with downstream enrichment steps to profile various types of PTMs *from the same sample sets*. The initial sample lysis, cleanup, and protein digestion steps are especially crucial for the success of an integrative approach because multiple PTMs of interest must be preserved during these processes. This is usually achieved by adding inhibitors (*e.g.*, phosphatase inhibitors for phosphorylation) or chemicals that “freeze” the endogenous PTM status (*e.g.*, *N*-ethylmaleimide (NEM) for alkylating cysteine free thiols). Thus, a universal sample preparation method should be compatible with a spectrum of chemicals and effective at sample cleanup, so that the resulting peptides are contaminant-free for enrichment and LC-MS/MS. In addition, ideal approaches must be flexible for various protein inputs (sub micro- to milligram quantities) and sample types, provide high protein recovery in an unbiased fashion, and be amenable to automation for high-throughput processing.

Single-pot, solid-phase–enhanced sample preparation (SP3) satisfies many of the requirements of a cost-effective high-throughput integrative proteomics approach (35). It boasts a wide range of chemical compatibilities including detergents for promoting cell lysis, solubilizing hydrophobic membrane proteins, and denaturing proteins (36, 37). Moreover, it performs markedly better at recovering quantity-limited samples and provides drastic improvements in digestion efficiency, especially when compared to in-solution methods like acetone precipitation and chloroform/methanol precipitation (35, 38, 39, 40). SP3 employs carboxylate-functionalized magnetic beads to capture solvent-aggregated proteins and, thus, can be easily automated (17, 41). This was exemplified by Leutert *et al*. who developed an automated approach for sample processing and subsequent phosphopeptide enrichment on the KingFisher magnetic particle handling platform (17). SP3 has also been used in chemoproteomics profiling of reactive cysteines and were labeled with iodoacetamide alkyne and then biotinylated for enrichment *via* copper-catalyzed click chemistry (42, 43, 44).

**Fig. 1:**
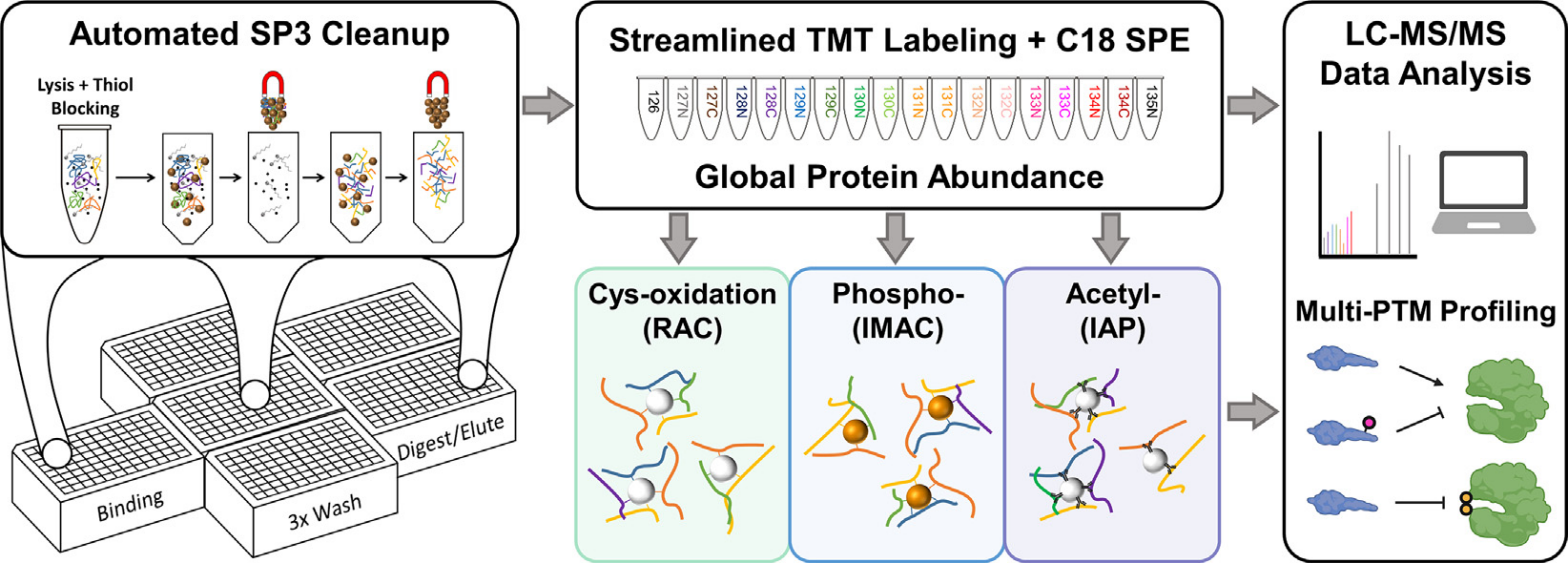
**Overview of the integrative workflow for multi-PTM profiling.** Following lysis and blocking free cysteine thiols with NEM, samples are processed in an automated fashion using SP3 beads. The resulting peptides are then labeled with TMT reagents in a streamlined step that permits immediate desalting *via* C18 SPE. The pooled samples are split for multiplexed quantification of global protein abundance and downstream enrichment steps to quantify cysteine thiol oxidation, phosphorylation, and acetylation.

In this work, we developed an integrative and automated SP3-based workflow for the quantification of multi-PTMs (Fig. 1). We first adapted the SP3 method to our previously established redox proteomics workflow that uses acetone precipitation and in-solution digestion (referred as acetone precipitation workflow) for sample preparation (45, 46). We demonstrate that SP3 vastly simplifies the analytical workflow by eliminating the laborious and time-consuming washing and buffer exchange steps in the acetone precipitation workflow. The SP3 workflow generates tryptic digests that are readily compatible with the enrichment of peptides bearing phosphorylation, cysteine thiol oxidation, or acetylation. To improve throughput, we automated SP3 on KingFisher and incorporated tandem mass tag (TMT) multiplexing to substantially reduce sample count (47, 48). The pooled, TMT-labeled peptides are then split for global quantification and PTM enrichments. Thiol-affinity resin-assisted capture (RAC) is used to enrich cysteine-containing peptides, which covalently bind to the resin and thus lead to a much higher enrichment selectivity than biotinylation-based enrichment (49). For phosphorylation, we incorporate an immobilized metal affinity chromatography (IMAC)-based enrichment workflow, the flowthrough of which can also be used for serial enrichment of acetylation (acetyl-) (34). The integrative SP3-based workflow was tested in both cell and tissue samples and permits a large range of protein inputs. The utility of this approach for profiling multi-PTM dynamics and interplay was further demonstrated in a time-course experiment of pancreatic β-cells treated with inflammatory cytokines.

## Experimental Procedures

### Growth and Lysis of Cell and Tissue Samples

Three biological systems were used in this study: a nontumorigenic alveolar type II epithelial cell line C10 (graciously provided by Dr Galya Orr), BALB/C mouse gastrocnemius muscle tissue (BIOIVT), and an insulinoma-derived cell line β-TC-6 (ATCC; CRL-3605). In all systems, parallel workflows were conducted to analyze reversible cysteine thiol oxidation (endogenous-free thiols blocked with NEM) and total thiol content (NEM blocking omitted; Fig. 1*A*), and precautions taken to preserve the redox status are detailed below. Details on blocking endogenous free thiols using NEM are provided for C10 and β-TC-6 cells below. Muscle tissue homogenization was performed according to Day *et al*. (45).

C10 cells were cultured as described previously (50). After reaching full confluency, plates were trypsinized using 3 ml of 0.25% trypsin EDTA solution (Thermo Fisher Scientific) and centrifuged at room temperature for 4 min at 13,000 rpm. Cell pellets were washed twice using 10 ml of cold PBS with or without 100 mM NEM and centrifuged at 13,000 rpm and 4 °C for 4 min. For analysis of phosphorylation, 10 mM NaF (Sigma Aldrich) and 1% Phosphatase Inhibitor Cocktails 2 and 3 (Sigma Aldrich) were additionally added to PBS prior to collection and washing. Stocks of about 1 × 10^6^ cells were aliquoted, snap-frozen in liquid nitrogen, and stored at −80 °C. For cell lysis, tubes were thawed on ice and 200 μl of lysis buffer containing 250 mM MES (pH 6.0), 5% SDS, and 1% Triton X-100 was added (with or without 100 mM NEM). To avoid detergent precipitation, samples were immediately lysed, and DNA was sheered using a probe sonicator (Fisher Scientific Series 60 Sonic Dismembrator) with 3 × cycles of 15 s sonication followed by 20 s ice incubation. Samples were then centrifuged at 13,000 rpm and 4 °C for 4 min. Protein concentration of cell lysate was quantification by the BCA assay (Pierce). Samples were incubated in a thermomixer (Eppendorf) at 55 °C, 850 rpm for 30 min to denature proteins for efficient NEM alkylation.

β-TC-6 cells were cultured in RPMI-1640 with 11.1 mM glucose (Gibco), 15% FBS (Gibco), 1% glutamine (Glutamax), and 1% antibiotic-antimycotic (Gibco, cat. #15240062). Cells were plated in 100 mm culture dishes at ∼80% confluency 24 h prior to treatment. For cytokine treatment, culture media was removed and replaced with 8 ml of fresh media containing a cytokine cocktail (100 U/ml interleukin-1 beta (IL-1β) + 250 U/ml interferon gamma (IFN-γ); both supplied by Peprotech) (51). After cells were incubated for the desired amount of time (4, 8, and 24 h), the cytokine-containing media were removed, and cells were rinsed twice with 5 ml cold PBS with or without 100 mM NEM for the analysis of thiol oxidation or total thiol content, respectively. After the last rinse, residual PBS was aspirated, and culture dishes were immediately placed at −80 °C until further processing. Lysis and NEM alkylation were performed in the same manner as with the C10 cells.

### Manual SP3 Workflow for Proteomics Sample Preparation

Sera-Mag SpeedBead Carboxylate-Modified E3 (GE Healthcare, cat. #65152105050250, 50 μg/μl, hydrophobic) and E7 (GE Healthcare, cat. #45152105050250, 50 μg/μl, hydrophilic) were prewashed and prepared according to guidelines from Cytiva (see Supplemental Methods). SP3 was performed with a bead/protein ratio of 10:1 (wt/wt) for 200 μg protein inputs and 5:1 for 1 mg inputs (37).

For 200 μg protein inputs, the sample volume was adjusted to 200 μl and then 40 μl of the bead mixture was added. Samples were incubated for 5 min at room temperature, 1000 rpm shaking. Next, 400 μl of absolute molecular grade ethanol was added. After incubation for 5 min at room temperature and 1000 rpm, samples were placed on the magnetic rack for 2 min. The supernatant was removed and the beads were washed twice using 500 μl of 80% ethanol (2 min at room temperature with shaking at 1000 rpm). After the final wash, residual ethanol was carefully removed, and the beads were pelleted *via* brief tabletop centrifugation. On-bead digestion was performed by incubating the samples in 100 μl of digest mix (proteases dissolved in 50 mM Hepes, pH 7.7) at 37 °C with 850 rpm shaking overnight. Initial experiments tested the type of proteases and the protease/protein ratio (wt/wt), and an optimized digestion condition with trypsin (Promega) and Lys-C (Wako Chemicals) both at 1:100 ratios was used. After digestion, samples were placed on a magnetic rack for 2 min and the eluate was transferred to a new tube. A second elution was performed by adding 100 μl of 250 mM Hepes (pH 7.7) with 5% acetonitrile (ACN) and shaking the samples at 850 rpm for 2 min. The two elutions were combined and placed on the magnetic rack for another 2 min to remove residual beads. Finally, the liquid was transferred to a new tube. SP3-derived peptides were subjected to C18 desalting for label-free analyses or TMT labeling for multiplexing experiments. Desalting was performed using solid phase extraction (SPE; Sep-Pak, 50 mg C18 columns) and the cleaned peptides were dried *via* vacuum centrifugation using a speedvac.

For 1 mg protein inputs, a similar procedure was used with several adjustments. First, 100 μl of bead mixture was transferred to tubes, which were then placed on the magnetic rack for 2 min to remove the supernatant. Second, 500 μl of samples containing 1 mg protein was added to the tubes, to which 500 μl of absolute ethanol was added to induce binding. Third, five washes were performed with 1 ml of 80% ethanol. Lastly, 500 μl of digest mix was used for digestion and 500 μl of 250 mM Hepes (pH 7.7) with 5% ACN was used for the second elution.

### Automated Sample Cleanup for Proteomics Sample Preparation Using SP3 Beads

Automation was performed on KingFisher Flex (Thermo Scientific) according to Leutert *et al*. with slight modifications (17). For 200 μg protein inputs, the sample volume was adjusted to 200 μl in deep-well plates, and 400 μl of absolute ethanol was added (sample/binding plate). The bead plate was prepared by transferring 40 μl of the 50 μg/μl washed beads to 160 μl water in their respective wells (10 μg/μl final). In addition, three wash plates containing 600 μl of 80% ethanol were also prepared. The comb, bead, sample/binding, and wash plates were loaded onto KingFisher, and the program was started to transfer the beads to the sample/binding plate, followed by incubation, and washing. During the washing steps, the digestion plate was prepared with each well containing 100 μl of digestion mixture (1:100 trypsin and 1:100 LysC in 50 mM Hepes buffer, pH 7.7). After washing, the beads were transferred to the digestion plate and digestion was performed offline on an incubator (3 h at 37 °C with 500 rpm shaking). Near the end of the digestion step, 100 μl of 250 mM Hepes (pH 7.7) with 5% ACN was added to wells of the second elution plate, which was loaded onto KingFisher. After digestion, the digestion plate was returned to the KingFisher, and the program was resumed to transfer beads to the second elution plate and perform brief mixing. Both the digestion plate and second elution plate were removed, and beads in the second elution plate were separated on a MagnaBot FLEX 96 magnetic plate (Promega). Finally, the supernatants from the digestion plate and second elution plate were combined. For multiplexed quantification, 100 or 200 μg of eluted peptides were labeled with TMTpro 18-plex (Thermo Scientific) in a 2.5:1 mass ratio of label:peptides and quenched with 5% hydroxylamine. Afterwards, the samples were desalted using C18 SPE (Sep-Pak, 50 mg columns) and dried in a speedvac. Note that TMT labeling schemes include empty (“skipped”) TMT channels (*e.g.*, in Supplemental Fig. S6*B*) because total thiol (−NEM) channels yield more peptides after RAC enrichment and, thus, higher contaminating isotope intensities that would otherwise bleed over to the lower intensity total oxidation (+NEM) channels.

For 1 mg protein inputs, additional modifications were implemented to maximize bead transfer and accommodate the higher protein loads. Sample volume was adjusted to 500 μl in the sample/binding plate, and 500 μl of absolute molecular grade ethanol was added. The bead plate was prepared by transferring 100 μl of the 50 μg/μl washed beads to 100 μl water in the respective wells (25 μg/μl final). The three wash plates contained 1 ml of 80% ethanol. After the beads were released to the first wash plate, an additional step was added to the program such that the magnetic comb returns to the sample/binding plate to collect residual beads and transfer those to the first wash plate. The digestion plate was prepared with each well containing 500 μl of digestion mixture, and the second elution plate contained 500 μl of 250 mM Hepes (pH 7.7) with 5% ACN. Digest and elution steps were conducted in the same fashion.

### Resin-Assisted Capture for the Enrichment of Cysteine-Containing Peptides

Synthesis of the thiolated sepharose resin is described in a recent publication from our group (45). Peptide-level RAC of cysteine-containing peptides was conducted similarly to a previously reported workflow (45, 46, 49, 52). In summary, 100 to 200 μg peptides derived from SP3 + C18 SPE were reduced in 5 mM DTT then the volume was increased to bring the DTT concentration down to 0.8 mM. Each sample was incubated with 30 mg of pre-washed resin followed by extensive washing and multiple elutions containing 20 mM DTT (52). Samples were dried in a speedvac then desalted using Zip-Tip C18 SPE (Omix) for label-free samples. For TMT-labeled samples containing total thiol booster channel(s), the pooled samples were desalted with C18 SPE columns (Sep-Pak, 50 mg) and optionally fractionated *via* high-pH RPLC before LC-MS/MS.

### Immobilized Metal Affinity Chromatography for Enrichment of Phosphopeptides

Manual phosphopeptide enrichment was performed (without fractionation) as previously described using 400 μg peptides derived from SP3 + C18 SPE (53). For the β-TC-6 experiment, 1 mg inputs were fractionated according to Cuesta *et al*. (53), and phosphopeptides in the concatenated fractions were enriched using the automated method described by Abelin *et al*. (34).

### Automated Acetyl-lysine Immunoaffinity Enrichment

Acetylated lysine peptides were enriched with five μL of PTMScan Acetyl-Lysine Motif [Ac-K] immunoaffinity magnetic bead slurry with proprietary antibody amounts (PTMScan Acetyl-Lysine Motif Kit, Cell Signaling Technologies #46784) using KingFisher Magnetic Bead Processor (ThermoFisher Scientific). Phosphopeptide-depleted IMAC flowthroughs were concatenated from six to four fractions and dried down in Speedvac. Before enrichment, antibody beads were washed four times with ice-cold 1× PBS and resuspended in 1× immunoaffinity precipitation (IAP) bind buffer (Cell Signaling Technology, #25144). Peptides were reconstituted with 250 μL of 1× IAP bind buffer containing 0.01% CHAPS, added to washed immunoaffinity beads, and incubated for 3 h at 4 °C on an end-over rotator. Bead-bound acetyl-enriched peptides were washed four times with 1× HS IAP wash buffer (Cell Signaling Technology, #42424), 0.01% CHAPS, followed by one wash with ultrapure H_2_O with 0.01% CHAPS and elution with 100 μl of 0.15% TFA. Eluent was desalted using C18 stage tips, eluted with 50% ACN/0.1% formic acid (FA) directly into ALS vials and dried down. Acetylated peptides were reconstituted in 12 μl of 3% ACN/0.1% FA/0.04% *N*-Dodecyl-β-maltoside, and 5 μl were injected for LC-MS/MS analysis.

### High-pH Reverse Phase Capillary LC Fractionation

Microscale LC fractionation was used for deep profiling of global and redox peptide samples using an established setup (the corresponding data are presented in Supplemental Fig. S7 and Fig. 5) (11, 45). Thirty micrograms of peptides were fractionated using a reversed-phase LC column (65 cm × 200 μM internal diameter packed with 3 μm Phenomenex Jupiter C18 particles) on a nanoAcquity LC system (Waters) with a flow rate of 2.2 μl/min. The binary solvent system was comprised of mobile phase A (10 mM ammonium formate in water) and mobile phase B (ACN) at pH 7.0. Eluted peptides were concatenated into 12 fractions using an autosampler (HTC PAL; CTC analytics) running Chronos software. Fractions were dispensed into vials containing 20 μl of 0.01% *N*-Dodecyl-β-maltoside, dried, and resuspended in 20 μl of ultrapure water containing 20 mM DTT for final LC-MS/MS analysis.

### LC-MS/MS Analysis

**Fig. 2:**
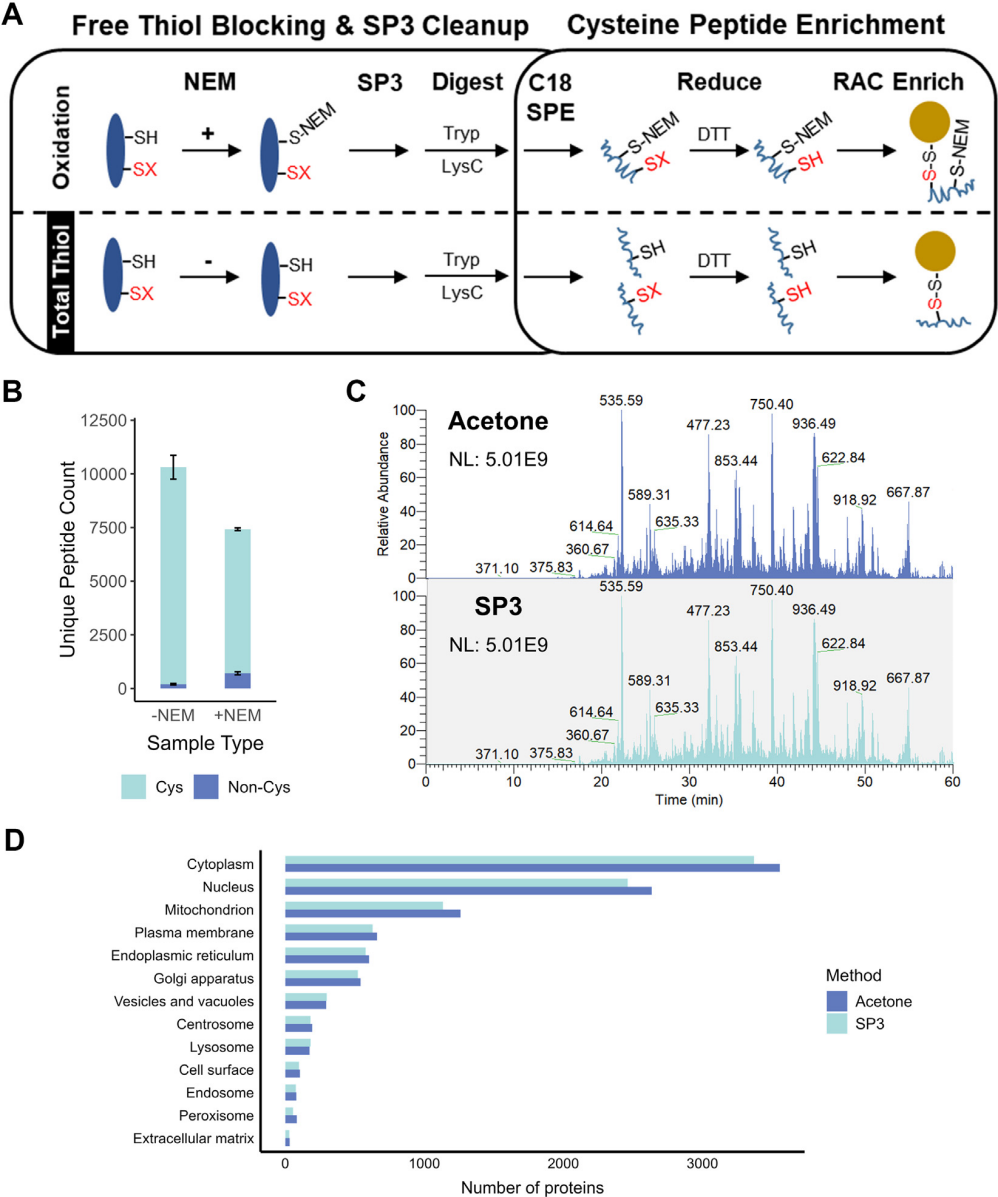
**Label-free SP3 approach for redox profiling.***A*, workflow schematic depicting sample preparation (day 1) and enrichment (day 2). During cell harvesting and lysis, protein-free thiols in “Oxidation” samples are blocked with NEM, whereas NEM is omitted for “Total Thiol” samples that are processed in parallel. SP3 is then used for sample cleanup and is followed by on-bead overnight digestion and additional C18 SPE cleanup. Reversibly oxidized cysteine thiols (represented by “-SX” in *red*) are reduced and enriched by resin-assisted capture (RAC). *B*, stacked bar chart showing the coverage attained using the SP3-RAC workflow. –NEM corresponds to “Total Thiol” samples while + NEM corresponds to “Thiol Oxidation” samples. Error bars are SDs of duplicates. *C*, total ion chromatograms for Total Thiol samples derived from RAC enrichment of cysteine-containing peptides. For sample preparation, SP3 was compared to the previously established acetone precipitation method (45). *D*, bar chart summarizing protein coverage in subcellular compartments (according to refined GO cellular compartment terms) from the acetone precipitation method *versus* SP3 (145).

**Fig. 3:**
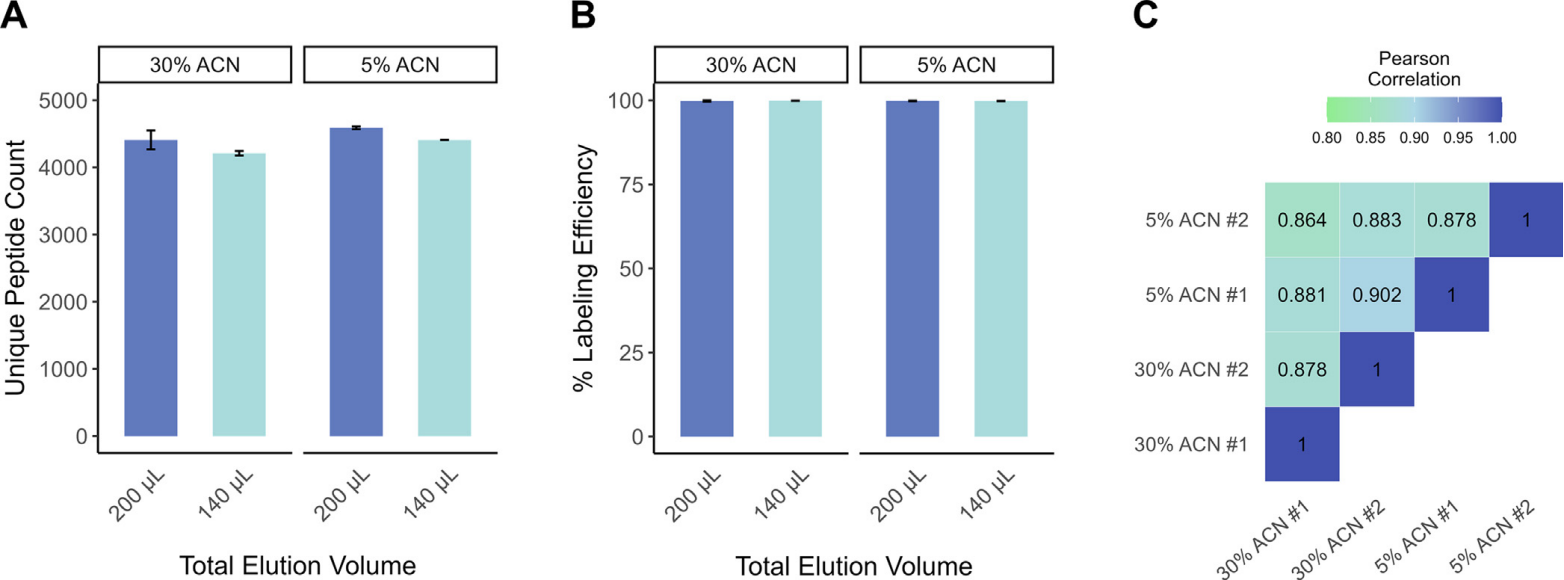
**Tuning the SP3 elution and TMT labeling steps.***A*, bar chart comparing peptide identifications with different SP3 elution volumes and vol% acetonitrile (% ACN) during TMT labeling. Total elution volumes are the sum of two equivolumetric elutions (*i.e.*, 200 μl = 100 μl digestion + 100 μl elution with buffer). % ACN does not include contribution from adding TMT labels, which increases the total vol% to about 11%. Error bars are SDs of duplicates. *B*, bar chart demonstrating the TMT labeling efficiencies. *C*, correlogram of protein abundances (duplicates) from the 200 μl elution condition.

**Fig. 4:**
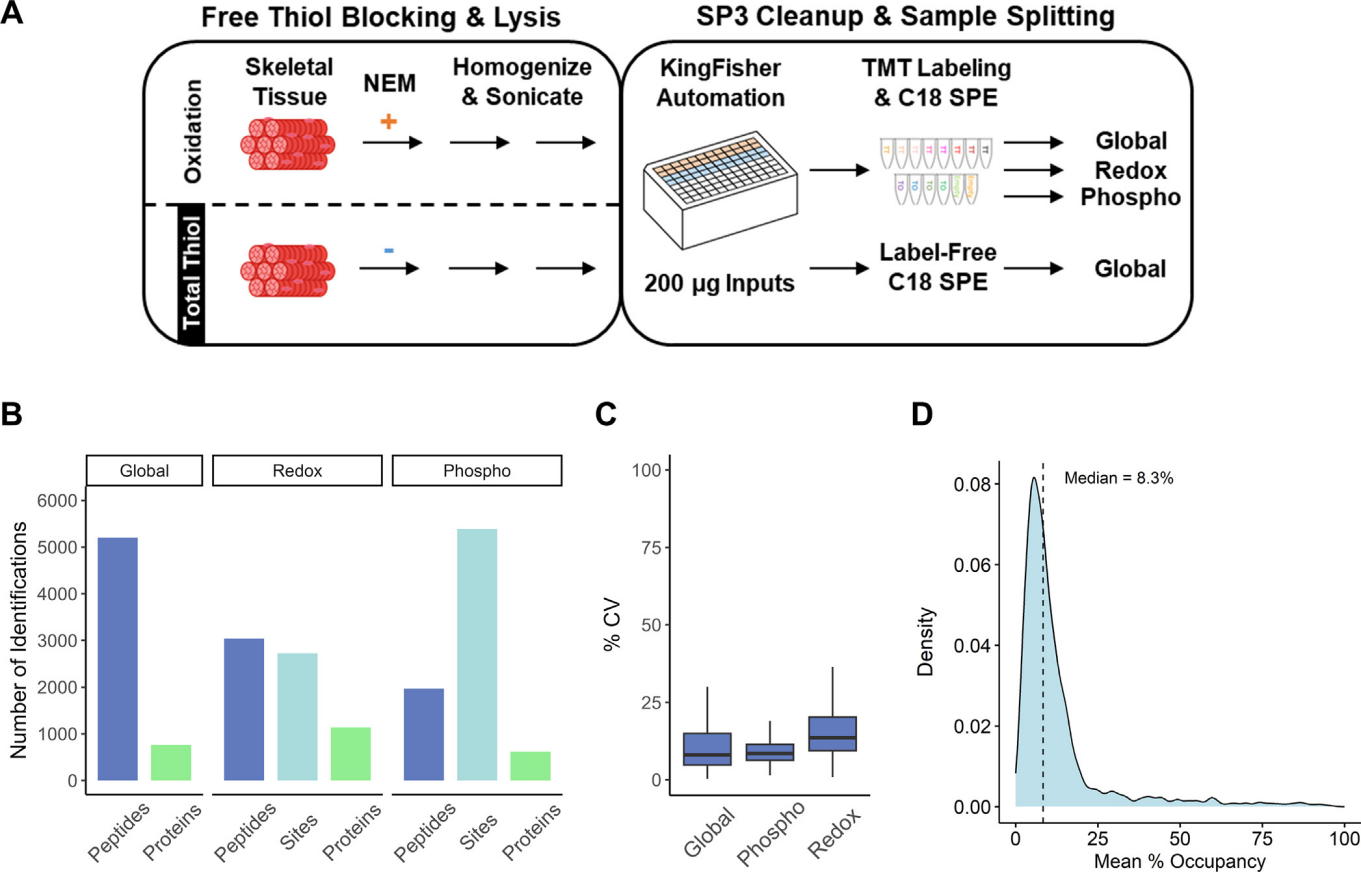
**Automated SP3 for redox and phosphoproteomics.***A*, the general workflow employed for SP3-RAC/IMAC. Briefly, muscle tissue was incubated with and without NEM, then lysed by homogenization followed by sonication to sheer DNA. Two hundred micrograms of proteins were used for automated SP3, and hundred micrograms of digested peptides were then used for each TMT channel (with eight Thiol oxidation samples and four total Thiol samples, Supplemental Fig. S6*B*). The pooled TMT set was desalted *via* C18 SPE, and the resulting eluate was split for global, redox, and phosphoproteomics. *B*, faceted bar chart displaying unique identifications from single-shot LC-MS/MS analysis of the split SP3-RAC/IMAC samples. *C*, box plots showing the distribution of peptide level CVs following median normalization. *D*, distribution of mean % cysteine thiol oxidation for muscle tissue.

Peptides were analyzed on an ACQUITY UPLC (Waters) coupled with Q Exactive Plus mass spectrometer (Thermo Scientific) or a Vanquish Neo UHPLC coupled with Orbitrap Exploris 480. Peptides were separated on a self-packed reverse-phase column (25 cm × 75 μm ID packed with Waters 1.7 μm BEH C18 material) with an integrated PicoTip emitter (New Objective). A binary mobile phase comprised of water with 0.1% FA and ACN with 0.1% FA was used. 1 h LC gradients were used for manual SP3 method development (data presented in Figs. 2, 3, and Supplemental Fig. S4), whereas 2 h LC gradients were used for separation of phosphopeptides, automation method development, and separation of fractionated samples (data presented in Fig. 4 and Supplemental Figs. S6–S8). Data-dependent acquisition was used with the following settings: full MS scans (m/z 300–1800) were collected at a resolution of 70,000 with maximum ion injection time of 20 ms and automatic gain control of 3E6. Fragment ion spectra were collected for up to 12 of the most abundant precursor ions. Precursor ions were first selected (isolation window of 1.5 m/z) and fragmented by higher collisional energy–induced dissociation at 30% normalized collisional energy. MS/MS scans were acquired at a resolution of 17,500 with maximum ion injection time of 50 ms and automatic gain control of 1E5. Fixed first mass was set to 110 m/z to include TMT reporter ions, and dynamic exclusion was 30 s.

### Data Analysis and Processing

LC-MS/MS data were searched using MS-GF+ (54) against *Mus musculus* protein sequences from UniProt (Release 2019-9 with 17,001 proteins for all analyses except those conducted for the β-TC-6 experiment, which used Release 2023-3 with 21,949 proteins) (55). A parent ion mass tolerance of 20 ppm and partial tryptic rule with up to two missed cleavages were the key searching parameters. Oxidation on methionine (+15.9949) and NEM blocking of cysteine (+125.047679) were selected as dynamic modifications. Phosphorylation of serine, threonine, and tyrosine residues (+79.9663) was also set as a dynamic modification when appropriate. The same searching parameters were used in MaxQuant (56) data processing for label-free quantification with a 0.5 Da fragment ion mass tolerance. For multiplexed quantification, TMT labels on peptide N-termini and lysine (+304.207146) were selected as fixed modifications. Lysine acetylation was set as a dynamic modification by calculating the mass difference between a fixed TMT label and an acetyl group (−262.196586). TMT labeling efficiency was determined using global samples with labeling set as a dynamic modification. Peptide spectra matches were filtered using the following criteria: 1) mass accuracy within 10 ppm; 2) PepQ value < 0.01 to a final false-discovery rate <1%. TMT reporter ion intensities were extracted by MASIC (57). For all analyses except the β-cell experiment, details regarding data analysis and processing using an in-house RStudio script are provided by Day *et al*. (45). Mean % cysteine thiol oxidation was calculated by averaging the normalized raw intensities of thiol oxidation channels and, separately, the TMT channels corresponding to total thiol samples. For each site, the average thiol oxidation intensity was then divided by the respective total thiol intensity.

For the β-cell experiment, the RStudio package “PlexedPiper” [https://github.com/PNNL-Comp-Mass-Spec/PlexedPiper] was used to merge MS-GF+ and MASIC data for the post-processing of TMT reporter ions to perform isobaric quantification (58). These were then processed using a custom pipeline for relative quantification of protein abundance, cysteine oxidation (“Redox”), phosphorylation (“Phospho”), and acetylation (“Acetyl”). Firstly, for global protein abundance data, peptide TMT reporter ion intensities were aggregated to the unique protein level by summing up raw reporter ion intensities of corresponding peptides. These data were then log_2_ transformed, batch corrected for multiple plexes, and median centered for normalization. Secondly, for PTM abundance data, peptide TMT reporter ion intensities were aggregated to the unique site level. The log_2_-transformed global peptide data (not batch corrected) was used to scale the site level PTM abundances to account for TMT channel loadings and for median centered normalization. These PTM data were then batch corrected. Finally, the average normalized protein abundances (per condition) were subtracted from the corresponding PTM site level abundances. IDs with more than two NA values per condition (2/4 missing values) were removed prior to statistical tests. Refer to Supplemental Table S10 for additional details.

The RStudio packages “limma” (59) and MSnSet.utils [https://github.com/PNNL-Comp-Mass-Spec/MSnSet.utils] were used for two-sample t-tests and one-way ANOVAs, respectively. The RStudio package ggpmisc was used to generate the best-fit line and equation shown in Supplemental Fig. S5*D* [https://docs.r4photobiology.info/ggpmisc]. KEGG (60) pathway over-representation analyses were performed using the “clusterProfiler” package (61). Heatmaps were made using the package “ComplexHeatmap” (62). All other figures were made with the “ggplot2” package (63). For structural investigations, we extracted active site residue numbers and AlphaFold2-predicted protein structures from the UniProt database using our in-house Python scripts (55, 64). Additionally, we utilized ChimeraX and a collection of data science tools (*i.e.*, Pandas, BioPandas, and Matplotlib) to visualize protein structures and calculate distances between specific residues of interest (65, 66, 67).

### Experimental Design and Statistical Rationale

The number of replicates is provided in each figure legend. Furthermore, TMT plex designs are presented for multiplexed quantification experiments (Supplemental Figs. S6–S9). For experimentation using C10 cells and muscle tissue, the same biological source was used following lysis with (or without) NEM to assess technical variability of the sample preparation methods. Database searching parameters and filtering thresholds for tandem mass spectra are detailed in the Data Analysis and Processing methods section. False discovery rate was estimated using the target-decoy approach (68, 69). Peptides quantified in at least two replicates were used for statistical analyses. The precision of quantification was analyzed using Pearson correlation with 95% confidence. % CVs were calculated using raw intensities, whereas two-sample t-tests were performed on log2 ratios, and the resulting *p*-values were adjusted for multiple comparisons using the Benjamini–Hochberg procedure. For the β-cell timecourse experiment, IDs from limma *t* test results with adjusted *p*-values ≤0.05 and absolute fold changes ≥0.8 were used for KEGG pathway over-representation analyses with enrichment results reported according to cutoffs of q-value ≤0.2 and adjusted *p*-value ≤0.05. The “4 h Control” condition was used as a reference for one-way ANOVA, and the results were filtered for adjusted *p*-values ≤0.05 and absolute fold changes ≥1 in at least one of the conditions to visualize protein and PTM abundance dynamics.

## Results

### A Redox Compatible SP3 Workflow

Our redox proteomics workflow employs an MES buffer at pH 6.0 during harvesting and lysis steps to minimize artificial thiol oxidation that occurs more frequently at neutral and alkaline pHs (45, 70, 71, 72). However, all SP3 redox and phosphoproteomics papers to date employ lysis buffers with pH ≥ 7 to satisfy recommended pH levels for protein binding to SP3 magnetic beads (17, 37, 42, 43, 73). We thus first tested the compatibility of our low-pH lysis buffer (250 mM MES, 1% SDS, and 1% Triton X-100, pH 6.0) with SP3 using bovine serum albumin. We found that the MES buffer system resulted in less loss during the protein binding step (<10%) compared to that of a published high pH buffer (100 mM Tris, 8 M urea, and 150 mM NaCl, pH 8.2; ∼20% loss during binding; Supplemental Fig. S1*A*) (17). In addition to a low-pH buffer system, our redox proteomics workflow uses a high concentration of NEM due to its fast reaction kinetics with cysteine and effectiveness in blocking free thiols at lower pH (42, 43, 74). Thus, we next tested the compatibility of 100 mM NEM with SP3 using bovine serum albumin. The bound proteins were digested and then eluted off the beads, which showed comparable peptide yields with or without 100 mM NEM (Supplemental Fig. S1*B*). We then tested the low-pH MES buffer containing NEM using the lysate from a mammalian cell line (mouse lung alveolar type II epithelial cells or C10 cells). Again, we found high binding between proteins extracted in the low-pH buffer and SP3 beads without the need to raise the pH and no interference of NEM for SP3 (Supplemental Fig. S2).

In SP3-based workflows, proteins are bound to the beads, washed, and digested on the magnetic beads. To enhance digestion, we evaluated various ratios of trypsin with or without LysC. Monitoring the eluted peptides using peptide quantification assays and SDS-PAGE showed high digestion efficiency (>50% yield) for all conditions tested (Supplemental Fig. S3). In addition, LC-MS/MS analysis showed similar numbers of unique peptide identifications between the trypsin-only and trypsin and LysC groups (Supplemental Fig. S4*A* and Supplemental Table S1). However, we found digestion with trypsin and LysC at enzyme-to-protein ratios of 1:100 for each resulted in <10% miscleavages, while ratios of 1:200 for each gave <15% miscleavages (Supplemental Fig. S4*B*). By contrast, >15% miscleavages were observed in trypsin-only groups. These data demonstrated that the SP3 workflow is compatible with a low-pH buffer containing 100 mM NEM and that on-beads digestion using both trypsin and LysC yields peptides with low levels of miscleavages.

With a modified SP3 workflow in hand, we then integrated RAC for the enrichment of cysteine-containing peptides (Fig. 2*A*) as a step towards developing a high-throughput method to quantify reversible cysteine thiol oxidation (10, 49, 75). In our approach, both “thiol oxidation” (all forms of reversible thiol oxidation combined) and “total thiol” (both reduced and reversibly oxidized thiols) are quantified. For oxidation samples, free thiols are first blocked with NEM, followed by SP3 cleanup/digestion and peptide cleanup, and reversibly oxidized cysteines are reduced and then enriched by covalently binding to a Thiopropyl Sepharose resin (49, 75). In parallel, “total thiol” samples are processed in the same manner, except that the initial NEM blocking step is omitted. As expected, LC-MS/MS analysis of the enriched samples showed more identifications for the total thiol (−NEM) samples than the thiol oxidation (+NEM) samples (Fig. 2*B* and Supplemental Table S2). In addition, the enrichment selectivities for both sample types were >90%. When compared to our previously established method using acetone precipitation, the SP3-RAC redox workflow affords similar MS results according to full MS1 total ion chromatograms (example shown in Fig. 2*C*). Moreover, there is no apparent biases for protein subcellular localization when comparing the two methods (Fig. 2*D*). However, a detailed comparison was not conducted given that acetone precipitation cannot be automated and that both methods are used for cleanup of detergent-containing lysates.

### Compatibility with Phosphopeptide Enrichment

After demonstrating that peptides derived from SP3 can be used for profiling thiol oxidation, we next confirmed that the modified SP3 workflow is compatible with phosphoproteomics. Because high concentrations of detergents in the lysis buffer denature proteins and inhibit phosphatase activity, phosphatase inhibitors were omitted from the modified SP3 workflow (76, 77). By comparison, a cocktail of phosphatase inhibitors was included in the lysis buffer for samples that were processed using acetone precipitation. IMAC enrichment was performed for both sample groups, and LC-MS/MS resulted in >15,000 unique phosphopeptide identifications for both approaches along with a similar profile of phosphorylated residues (Supplemental Fig. S5*A* and Supplemental Table S3). Moreover, the phosphopeptide enrichment selectivity was about 87% for both (Supplemental Fig. S5*B*). At the protein level, an overlap of ∼80% was observed among replicates (Supplemental Fig. S5), resulting in a total of 4300 quantifiable phosphorylated proteins. In addition, comparable LFQ intensities of phosphoproteins were observed between the two approaches (Supplemental Fig. S5*D*). The marked similarity between the approaches confirms the compatibility of the modified SP3 workflow with phosphoproteomics.

### Integrating and Streamlining TMT Labeling for Multiplexed Quantification

Multiplexing can drastically decrease the number of samples for enrichment and LC-MS/MS runs. In our TMT-based multiplexing workflow, peptides are labeled, pooled, desalted, and then split for parallel enrichment of different PTM types. Given the amount of SP3 beads used (2 mg for 200 μg protein input) per sample and the large sample multiplexing capacity afforded by TMT (*i.e.*, 18 plex at the time of writing), an elution volume of 200 μl per sample results in a large total volume after pooling. In addition, ACN is usually added to a final concentration of 30% for labeling to avoid hydrolysis of TMT (78, 79). However, high elution volumes and 30% ACN lead to a long wait time for speedvac drying of the pooled samples prior to C18 SPE. We thus tested a lower SP3 elution volume (140 μl) and lower ACN% for TMT labeling. We found that a lower total elution volume led to slightly lower unique peptide identifications at either 30% or 5% ACN (Fig. 3*A* and Supplemental Table S4). Excitingly, both 30% and 5% ACN facilitated comparable, high TMT labeling efficiencies (Fig. 3*B*). This lends support to other critical factors in TMT labeling including pH and the mass ratio between the TMT label and peptides (80). For proteins observed in all samples, Pearson correlation showed quantification coefficients between 0.86 to 0.90 (Fig. 3*C*). Because samples were not pooled in this experiment, the results represented interplex correlation, which was higher than the interplex correlation reported in literature (78, 79). The use of 5% ACN in labeling also allows one to omit the vacuum drying speedvac step and perform desalting directly after TMT labeling/pooling. This makes it possible to perform sample lysis, NEM blocking, SP3, TMT labeling, and RAC within the same day (Fig. 2*A*).

### Automated Sample Processing and Integrated Enrichments of Multiple PTMs

To make the SP3 sample preparation workflow more scalable, we implemented the method on a KingFisher magnetic beads handling system (17). Muscle tissue was used for the automation experiments to test its robustness and benchmark against our recent work that used the same tissue type with the acetone precipitation workflow for redox proteomics (45). Muscle tissue chunks were initially minced and then incubated in buffer with and without NEM, then homogenized and sonicated for lysis and DNA/RNA sheering, the latter of which can interfere with bead binding (Fig. 4*A*) (37, 40). Twenty four replicates for both unblocked and NEM blocked samples were processed in 96-well plates by KingFisher automation using SP3 beads. The automated method resulted in comparable peptide yields according to BCA, relative to those acquired using manual processing (Supplemental Figs. S3*A* and S6*A*). Following digestion and elution, randomly selected samples were TMT-labeled, pooled, processed by C18 SPE, and split for multi-PTM profiling (Fig. 4*A*; TMT labeling scheme in Supplemental Fig. S6*B*). For redox proteomics, we used peptide-level enrichment as described in recently published work (46, 81). QC metrics for single-shot LC-MS/MS runs showed a high TMT labeling efficiency of 99.3% and high enrichment selectivity for both redox and phosphorylation (91.4% and 86.8%, respectively). In addition, the unique identifications afforded by our automated method were very similar to those acquired using acetone precipitation and in-solution digest (Fig. 4*B* and Supplemental Table S5) (45). The automated method showed satisfactory quantification precision with <20% CV among replicates for all three data modalities (global, phosphorylation, and redox; Fig. 4*C*). To further assess quantification reproducibility, we also performed label-free proteomics using peptides generated by the automated sample preparation workflow. Both TMT-based and label-free assays yielded high Pearson correlations (>0.98, Supplemental Fig. S6, *C* and *D* and Supplemental Table S6). Lastly, automated sample processing with SP3 beads results in the expected mean % cysteine oxidation distribution (Fig. 4*D*) as compared to a recent publication that employed chloroform/methanol precipitation and in-solution digestion (81).

Deep profiling of various PTMs (including relatively rare types like acetylation) may require high protein inputs and/or serial enrichment steps that maximize the use of flowthroughs (34). Hitherto, experiments used 200 μg per sample; therefore, we implemented additional modifications (detailed in the methods section) to process 1 mg inputs on KingFisher. These modifications showed ∼50% peptide yields for 1 mg inputs, while improving the yield for samples of 200 μg input (Supplemental Fig. S7*A*). The resulting peptides from eight selected thiol oxidation and two total thiol samples were multiplexed in a TMT set and subjected to global and redox proteomics processing (Supplemental Fig. S7*B* and Supplemental Table S7). More than 5000 unique cysteine-containing peptides were identified with a single-shot LC-MS/MS run, while offline fractionation further increased the number to ∼20,000 (Supplemental Fig. S7*C*). Again, quantification precision was satisfactory with <20% CVs and high quantification correlations among replicates of the TMT set (Supplemental Fig. S7, *D* and *E*).

Since we processed 200 μg and 1 mg samples in parallel, we were also interested in comparing the quantification reproducibility among the two groups. The same peptide mass (500 ng on-column injection) was loaded for label-free analysis: high Pearson correlation coefficients were observed between the 200 μg and 1 mg sample groups (Supplemental Fig. S7*F* and Supplemental Table S8). These results demonstrate that the method can accommodate different inputs. In addition, we performed an indirect comparison between our automated *versus* manual SP3 workflows using 1 mg of muscle homogenates, which demonstrated the reproducibility of both methods (Supplemental Fig. S8*A* and Supplemental Table S9) and the similarity in total unique peptide and protein identifications for single-shot global proteomics samples (Supplemental Fig. S8, *B* and *C*).

### Application of the Multi-PTM Workflow in Pancreatic β-Cells Treated with Inflammatory Cytokines

**Fig. 5:**
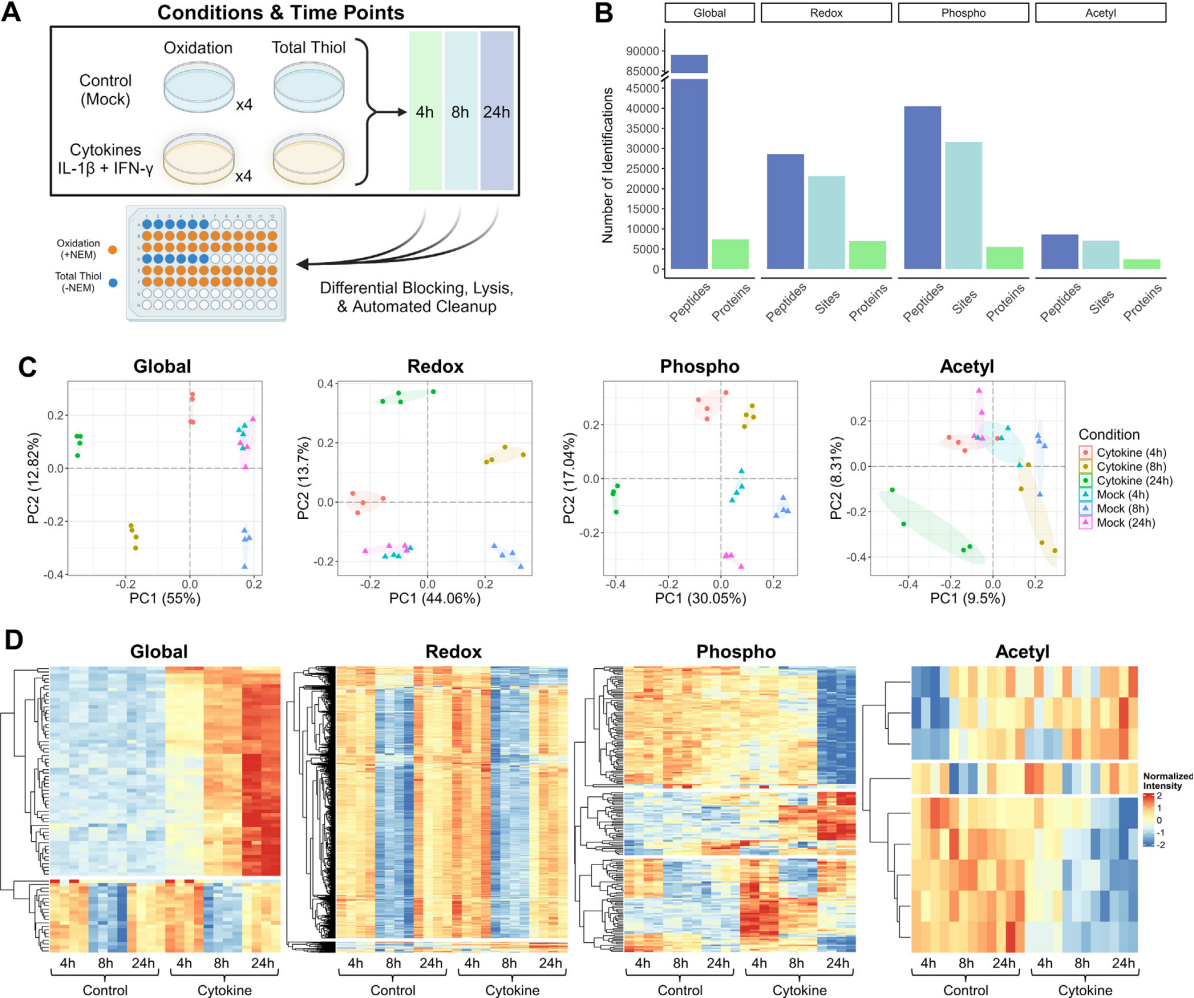
**Summary of multi-PTM profiling results from the β-cell timecourse experiment.***A*, experimental design. β-TC-6 cells were harvested from washed plates and differentially blocked with NEM (“Thiol Oxidation” in quadruplicate) and without NEM (“Total Thiol” in singlets). Cells were sonicated for lysis and then normalized to 1 mg inputs in technical duplicates for automated SP3. Only one set of the technical duplicates was labeled, pooled, and processed *via* C18 SPE (TMT labeling scheme in Supplemental Fig. S9). The pool of cleaned peptides was split for RAC, IMAC, and IAP to enrich cysteine-containing peptides, phosphopeptides, and acetylated peptides, respectively. Samples were fractionated prior to LC-MS/MS as described in the methods section. *B*, faceted bar chart showing unique identifications from fractionated samples. *C*, principal component analyses with ellipses delineating the 95% confidence intervals. *D*, ANOVA results presented in clustered heatmaps. The data was transposed and scaled to a mean of zero and SD of one prior to visualization (see the legend on the far *right*).

With the optimized multi-PTM profiling workflow, we next attempted to demonstrate its utility in profiling dynamic multi-PTM regulation in insulin-producing mouse pancreatic β-cells, the loss of which is a hallmark of type 1 diabetes (82, 83). β-cells (β-TC-6) were treated with IL-1β and IFN-γ, pro-inflammatory cytokines that induce β-cell apoptosis, for 4, 8, and 24 h according to literature (Fig. 5*A*) (51, 84, 85, 86). Samples from cytokine-treated cells and mock controls were multiplexed in two TMT18 plexes (Supplemental Fig. S9*A*) for relative quantification of global protein abundance, cysteine thiol oxidation, phosphorylation, and acetylation. Excitingly, our datasets covered over 7400 unique proteins, 23,000 Cys-sites, 31,500 phosphosites, and 7000 acetyl-sites (Fig. 5*B* and Supplemental Table S10). In terms of precision and reproducibility, our approach consistently resulted in CVs below 20% (Supplemental Fig. S9*B*).

Principal component analyses of the four omics modalities showed the separation of sample groups based on cytokine/mock treatment or time points (4, 8, and 24 h, Fig. 5*C*). For example, the global abundance data showed cytokine-treated samples were separated from mock-treated samples along PC1, while samples of each time point within the same treatment groups were separated on PC2. More complex patterns were observed for redox and phosphoproteomics samples, whereas a clear separation for the acetylomics samples was only observed for the cytokine treatment at 24 h (Fig. 5*C*). Because fresh medium was used for the cytokine/mock treatment, the Principal component analyses results suggested that the dynamics of protein expression and PTMs is a function of both cytokine treatment and cell adaptation. These trends were echoed in the heatmaps of significant proteins/PTMs that were differentially expressed among conditions (adjusted *p*-value <0.05 from one-way ANOVA, Fig. 5*D*). For instance, a cluster of proteins displayed a significant decrease in abundance at 8 h in both cytokine-treated and control samples. Given that cytokine treatment causes a significant change in protein abundance (even at 4 h), we normalized the PTM data against global protein abundances. The corrected redox data showed a similar decreasing trend at 8 h, though the effect was more pronounced than the global data. This contrasts with significant oxidation (and reduction) of select proteins clustering at the top and bottom of the redox heatmap. Notably, an increase in phosphorylation of many proteins was observed at 4 h, which remained high over the course of the experiment. For acetylation, significant changes were observed at the late time point 24 h. Volcano plots comparing cytokine-treated cells to time-matched controls showed similar trends (Supplemental Fig. S10). Overlapping of significant proteins/PTM sites (adjusted *p*-value <0.05) across the three time points also demonstrated time-dependent regulation with the most significant changes in global abundances occurring at 24 h and phosphorylation at 4 and 24 h (Supplemental Fig. S11). Thus, these data indicate that the level of protein abundance and PTMs varies according to a combination of cytokine treatment and the evolving environment, which includes important temporal considerations about nutrient availability and metabolic changes, cell adaptation, and activation of cell signaling pathways.

**Fig. 6:**
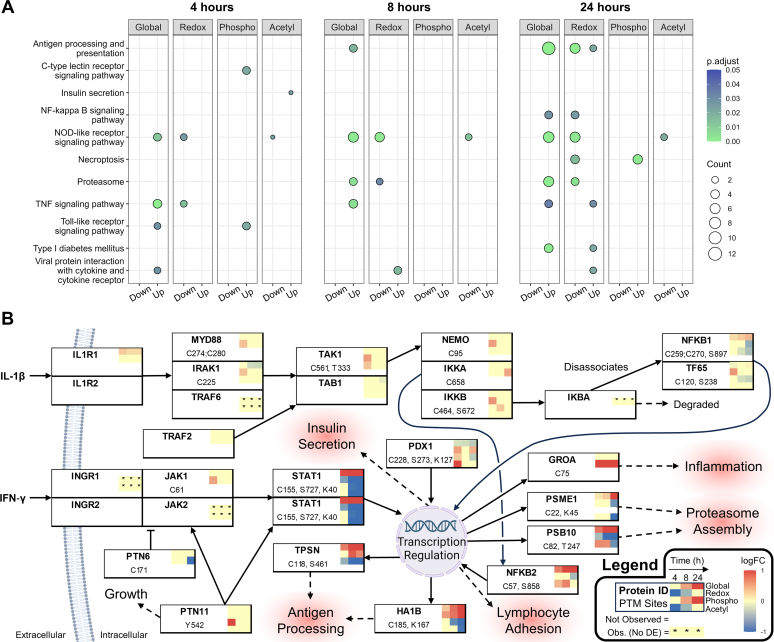
**Pathway enrichment results and representation of the NF-κB and JAK-STAT signaling pathways.***A*, faceted dot plots showing over-represented KEGG pathways. Adjusted *p*-value and q-value cutoffs of 0.05 and 0.2 were used for the pathway enrichment analyses. *B*, overview of the NF-κB and JAK-STAT signaling pathways that regulate inflammation, growth/cell cycle arrest, nutrient intake and insulin resistance, and proteasome assembly. The protein IDs in the boxes are gene nomenclature from UniProt. Grouped protein boxes represent complexes. Heatmaps show the mean log_2_FCs between cytokine and control for the four data modalities at 4, 8, and 24 h. *White* (empty square) designates no observations for that particular global or PTM dataset. PTM sites showing statistical significance were listed in the protein boxes, while the absence of sites indicates no significant differences among the observations. “Obs. (No DE)” refers to an observation for which not enough replicates were available after batch correction to perform differential expression (“DE”) or for which global protein data was not available for scaling PTM abundances. Note that a relative *decrease* in the occurrence of a particular PTM may be due to a parallel *increase* in protein abundance.

To further unravel the intricate cytokine/time-dependent PTM events, the results of differential expression analyses (adjusted *p*-value <0.05 and absolute log_2_FC > 0.8) were then used for KEGG over-representation analyses. As expected, several canonical signaling pathways involving antigen processing and presentation, NF-κB, TNF, and NOD-like receptor signaling pathway were enriched and are summarized in Figure 6*A* (87, 88, 89, 90). The dynamic trends discussed before were recapitulated: early signaling events are transduced by protein modifications which result in global changes to protein abundance, antigen processing and presentation, necroptosis, and cell cycle regulation at later time points. The complete dot plots revealed additional trends involving proteasome assembly and cAMP signaling pathway activity (Supplemental Figs. S12–S15).

### Integrated Proteomics Enables Dynamic Multi-PTM View of Cell Signaling Pathways

To demonstrate the power of our workflow in providing a dynamic multi-PTM view of cell signaling pathways, we next zoomed into the JAK-STAT and NF-κB pathways (Fig. 6*B* and Supplemental Fig. S16, which contains additional proteins and pathways of interest). JAK-STAT is a classical signaling pathway that mediates cellular responses to type II interferons (IFN-γ) by promoting antiviral gene expression, growth inhibition, and antigen processing and presentation (91, 92, 93, 94). Activation of JAKs requires phosphorylation of various tyrosine residues following cytokine-induced dimerization of the IFN-γ receptors. JAK1 and JAK2 then phosphorylate STAT1 at Y701, which results in STAT1 homodimerization nuclear translocation and regulation of target gene expression (91). STAT1 and STAT3 are phosphorylated at S727 in response to IFN-γ, but the role of STAT3 in this classical pathway is contentious (91, 92, 95, 96).

The rapidity of IFN-stimulated gene expression changes is supported by drastic upregulation of STAT1 at 4 h (log_2_FC = 0.97) and a steady increase in STAT3 (log_2_FC = 0.98) over 24 h (97). Because of a parallel decrease in STAT1 and STAT3 phosphorylation (Fig. 6*B* and Supplemental Fig. S16), it is likely that various phosphorylation events happened prior to the 4 h time point, which was reported in human-derived β-cells (98). The swift response of the JAK-STAT signaling pathway is strongly evidenced by the upregulation of several of its gene targets at 4 h. These include the antiviral GTPases GBP2 (log_2_FC = 1.36), GBP4 (log_2_FC = 1.12), GBP7 (log_2_FC = 1.39), IRGM1 (log_2_FC = 0.96), and IIGP1 (log_2_FC = 1.10) as well as proteins involved in antigen processing and presentation such as the ABC transporter TAP1 (log_2_FC = 0.83) and the MHC class I assembly protein tapasin (TPSN; log2FC = 0.71) (99, 100, 101, 102, 103). Activation of the tyrosine phosphatase SHP2 (encoded by PTPN11) is another early event that may lead to growth arrest *via* modulation of JAK-STAT, Ras, MAP kinase, and PI3 pathways (104, 105, 106, 107, 108). PTPN11 phosphatase activity requires phosphorylation on Y542, which increased dramatically at 4 h (log_2_FC = 1.07). PTPN11 can dephosphorylate JAKs and STATs, attenuating their growth inhibitory effects (107, 108).

In addition to phosphorylation, changes in the redox state of cysteine residues play important roles in cell signaling. We found a more reduced C171 (log_2_FC = −1.82 at 24 h) on tyrosine phosphatase SHP1 (encoded by PTPN6), which is implicated in epidermal growth factor receptor and β-cell receptor signaling and is particularly sensitive to oxidation (109, 110, 111). Another interesting example of cysteine oxidation was observed in the PI3K-AKT-mTOR pathway, which engages in direct crosstalk with JAKs and STATs to regulate vital cellular processes like proliferation (106, 112, 113). mTOR exhibited sustained oxidation of C1004 (max log_2_FC = 1.31 at 8 h), a residue in the HEAT repeats subdomain that is involved in mTOR dimerization and interactions with other proteins like Raptor and Rictor (55, 114). Although prior research shows that mTORC1 is redox-regulated, oxidation on C1004 has not been reported before.

In response to IL-1β and other activators, the NF-κB signaling pathway regulates the expression of pro-inflammatory genes and plays a salient role in cell survival and lymphocyte interactions (90, 115, 116, 117, 118). The IκB kinase (IKK) complex is a key regulator of NF-κB signaling and consists of kinases IKKα and IKKβ and a regulatory subunit NEMO (119). We found significant albeit small increases in cysteine oxidation for IKKα^C658^ (log_2_FC = 0.48 at 24 h), IKKβ^C464^ (log_2_FC = 0.58 at 4 h), and NEMO^C95^ (log_2_FC = 0.53 at 4 h) (Fig. 6*B*). Upon activation, the IKK complex phosphorylates the NF-κB inhibitor IκB (“IKBA” in Fig. 6*B*; “IKBB” also observed but not shown). IκB disassociates from the NF-κB complex consisting of NFKB1 (p50) and TF65 (RelA), thereby leading to its nuclear translocation (90). In our data, relatively small changes to the canonical NF-κB pathway enzymes were observed, while appreciable upregulation of NFKB2 in the noncanonical NF-κB pathway was (*e.g.*, log_2_FC = 1.09 at 8 h) (120). As with JAK-STAT, we observed significant upregulation of NF-κB signaling target genes at 4 h. These proteins include the CXC motif-containing chemokines CXCL9 (log_2_FC = 1.19) and CXCL10 (log_2_FC = 3.04) as well as ICAM1 (log_2_FC = 1.86), a transmembrane protein which stimulates lymphocyte interactions with β-cells (121, 122, 123, 124). Unlike the aforementioned chemotactic cytokines, CXCL1 (GROA) did not significantly increase in abundance; however, it had much higher and sustained cysteine oxidation (log_2_FC = 1.89 for C59 and 4.19 for C75 at 8 h). These cysteine residues form disulfide bonds and are crucial for the stabilization of active monomeric and dimeric forms of this protein (125, 126, 127).

Though upregulation of acetylation for proteins directly involved in the JAK-STAT and NF-κB signaling pathways was not observed, increases in acetylation were seen in proteins involved in gene expression regulation and insulin secretion (Fig. 6*B* and Supplemental Fig. S16). Our results showed acetylation dynamics for nucleolin NUCL^K9/16^ (log_2_FC = 1.06; adj. *p* = 0.07 at 4 h), the transcriptional coregulator HCFC1^K836^ (regulates PDX1 expression; log_2_FC = 1.03; adj. *p* = 0.07 at 4 h), and the pre-mRNA splicing factors ISY1^K121^ (log_2_FC = 1.10; adj. *p* = 0.07) and SFPQ^K551^ (log_2_FC = 1.296; adj. *p* = 0.07) (55). PDX1 is a major transcription factor that regulates expression of insulin and other proteins in the insulin secretion pathway (128, 129). It was slightly downregulated (log_2_FC = −0.34 at 24 h) in our results, but more intriguingly, acetylation of K127 was upregulated 1.13-fold at 4 h with no significant differences thereafter. This residue is adjacent to a peptide motif that mediates heterodimerization with PBX, an important complex for β-cell function (130, 131, 132). We also observed upregulation of insulin (INS2) acetylation at K88 (log_2_FC = 1.02 at 24 h), which to the best of our knowledge has not been reported. Overall, multi-PTM dynamics span multiple pathways highlighting the complexities of how β-cells respond to cytokine-induced stress and the necessity of a complete systems-level view of these phenomena to drive discoveries relevant to diabetes research.

### Leveraging Multi-PTM Dynamics and Protein Structures to Study PTM Interplay

**Fig. 7:**
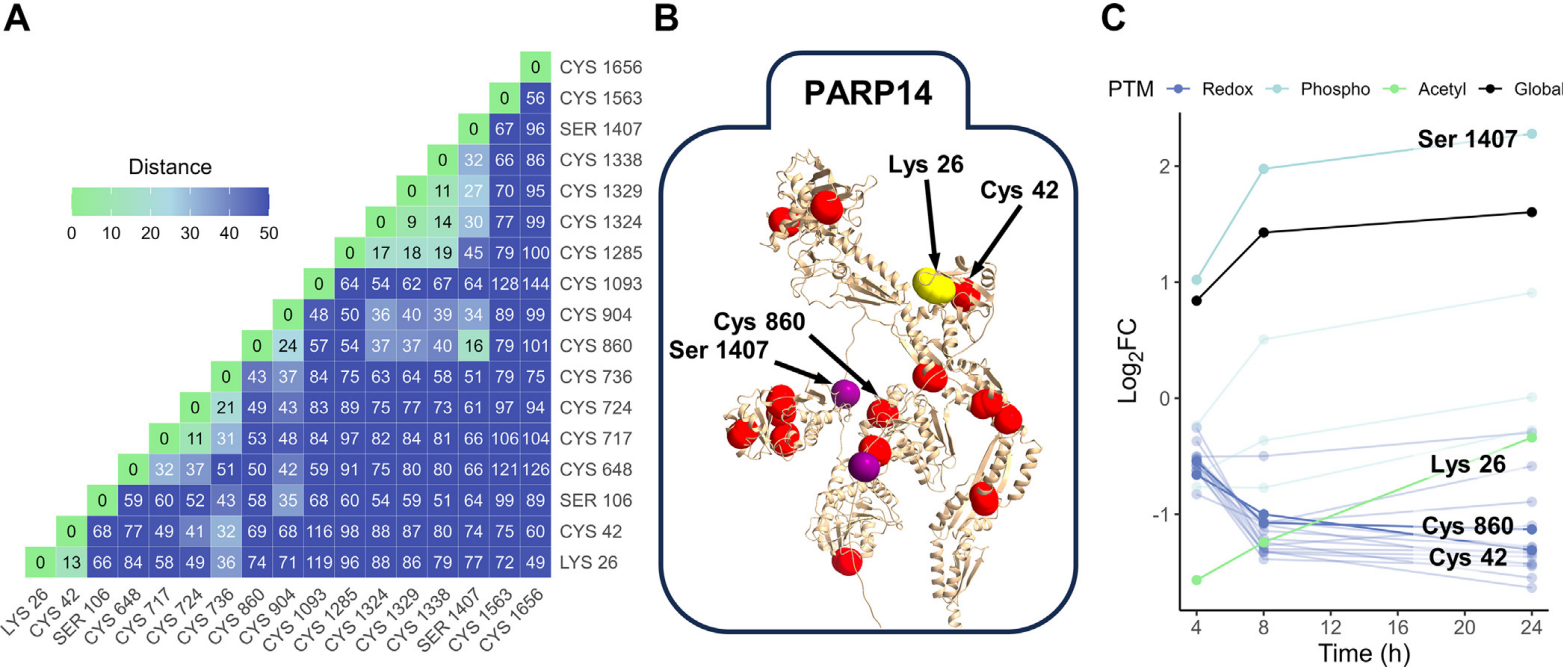
**Spatial mapping of differentially regulated PTM sites.***A*, heatmap depicting 3D Euclidean distances (in Å) between alpha carbons of select residues in PARP14. *B*, cropped image of the predicted PARP14 structure highlighting modified residues of interest. The colored residues denote the following: *red* = cysteine, *yellow* = lysine, and *purple* = serine, threonine, or tyrosine. *C*, line chart showing PTM (or cysteine site) log_2_FCs normalized to global protein abundances for PARP14. A decrease in log_2_FC for cysteine residues indicates a possibly more reduced thiol state. Log_2_FCs for global protein abundances are also included in *black*.

A structural perspective of multi-PTM localization is crucial for linking PTMs to protein regulation and function. We selected PARP14 to demonstrate the utility of mapping PTM data onto protein structures because it displayed multiple differentially regulated PTMs. To reveal the spatial distance among PTM sites, we generated heatmaps that depict 3D Euclidean distances (in Å) between alpha carbons of PTM residues from AlphaFold2 predicted structures (Fig. 7*A*). We found multiple PTM sites colocalize in intrinsically disordered regions with low AlphaFold2 prediction scores (Supplemental Fig. S17) (64). Spatial coregulation of multiple types of PTMs in the intrinsically disordered regions was investigated recently and lends support to our observations (133).

PARP14 is a mono-ADP-ribosyltransferase involved in DNA repair, transcriptional regulation, cell survival, and modulation of immune responses (134). It is a negative regulator of the JAK-STAT pathway, thereby affecting pro-inflammatory cytokine production, and a positive regulator of NF-κB signaling, which promotes apoptosis (135, 136). Outside of transcriptional activation, regulation of PARP14 activity is ill-defined, even though it is modified by various types of PTMs (137). Some of these sites may be colocalized and include K26 and C42 as well as C860 and S1407 (Fig. 7, *A* and *B*). Ubiquitylation of K26 is reported on PhosphoSitePlus, but acetylation is not and may be a newly observed modification (137). K26 and C42 are found in the RNA recognition motif domain 1 (134). C860 is found in macrodomain 1 which has ADP-ribosyl hydrolase activity (134). Interestingly, S1407 phosphorylation and K26 acetylation increased over time in the cytokine-treated cells (Fig. 7*C*). This trend was paralleled by a decrease in cysteine thiol oxidation suggesting the importance of maintaining PARP14 in a reduced redox state.

One of the caveats of using AlphaFold2 predicted protein structures is that some proteins or select regions therein have low prediction accuracies. For instance, AlphaFold2 indicated low prediction accuracy for the long middle loop of PARP14, which denotes structural flexibility (Supplemental Fig. S17). Additionally, given the absence of a stable hydrophobic core in PARP14, we anticipate that this conformation exists only transiently in nature. Therefore, we recommend careful interpretation of the reported distance information but maintain that this approach provides a largescale screening step for uprooting potentially colocalized PTMs to form hypotheses about PTM interplay. This approach also provides a foundation for studying larger questions about the structural context of redox PTMs and the coregulation of different PTM types.

## Discussion

Labor cost per processed sample is one of the most important metrics defining productivity. Manual sample processing workflows such as acetone precipitation and in-solution digest are time-consuming, susceptible to reproducibility issues, and impractical for large-scale deep multi-PTM profiling. These are the primary drivers for developing a fully automated, universal sample processing workflow. We demonstrate a modified workflow based on SP3 magnetic beads that streamlines cleanup, on-bead digestion, and TMT-labeling steps. Peptides generated using the universal workflow can be subsequently enriched in parallel for multi-PTM profiling. As proof of the integrative principle, we employed the workflow to study protein abundance, oxidation, phosphorylation, and acetylation in cytokine-treated β-cells. Our workflow allows two workers (6 h each) to lyse, cleanup, digest, and isobarically label 60 samples in a single day: (1) 4 h for lysis plus free thiol blocking, (2) 1.5 h for automated sample cleanup, (3) 2 h for TMT labeling, and (4) 1 h for C18-SPE followed by overnight vacuum drying. Of course, the method is flexible to overnight digests. The proceeding enrichments require 4 to 8 h and were completed in 3 days. By contrast, acetone precipitation followed by laborious protein resuspension requires 1.5 additional days of tedious work and labor costs to process that many samples. Our integrative SP3 workflow has several other attractive attributes such as high reproducibility, digestion efficiency, and downstream enrichment selectivity. As an important step forward for redox proteomics, our semi-automated workflow also yields expected % cysteine oxidation distributions (71, 138) and sets the stage for fully automated sample preparation.

The overarching impetus for this work was to establish a platform for the quantification of dynamically interacting regulatory pathways, which will provide large datasets to bioinformatically define PTM interplay. We successfully demonstrated our high-throughput platform through a time course experiment of cytokine-treated β-cells; however, we recognize the colossal challenges involved in designing experiments for multi-PTM profiling and interpreting the resulting deluge of data. Even in our model system, very little is known about the dynamics of different PTMs, and how protein abundance shifts affect the propagation thereof. For example, only minute (absolute log_2_FC < 0.8) differences in phosphorylation and cysteine oxidation were observed in the NF-κB pathway (Fig. 6*B*). At the very least, we expected to observe cysteine oxidation indicative of disulfide bond formation for NEMO dimerization (139). There are several possible explanations: the chosen time points did not capture the expected pathway perturbations, the NF-κB pathway is only weakly activated in β-cells (140) as a response to the specific cytokine cocktail and dose/cell count, the interactions among pathways activated by IL-1β and IFN-γ are difficult to interpret, and/or the coverage for this pathway was simply low (*e.g.*, IKBA, IKKA, MYD88, TRAF2, and TRAF6 in Fig. 6*B*). The first explanation seems the most reasonable because rapid upregulation of CXCL9 (log_2_FC = 1.19 at 4 h), PARP14 (log_2_FC = 0.84 at 4 h), NFKB2 (log_2_FC = 1.09 at 8 h), and others discussed above signifies a pro-inflammatory phenotype (98, 135).

Though a major focus of this study was to integrate our redox proteomics workflow with SP3, there are several challenges and drawbacks with our approach. One limitation is the lack of research regarding the potential effect(s) of NEM alkylation on the coverage of different PTMs. Another data analysis hurdle involves missingness due to batch effects (multiple TMT-plexes) that are compounded when scaling site-level PTM abundances to shifts in global protein abundances. In other words, a low abundance protein may be observed in the enriched samples but may not be in the global samples (*e.g.*, JAK2^Y570^ phosphorylation was observed but corresponding protein abundances in the global data were not). More work will be required to optimize data analysis pipelines for multi-PTM profiling and improve accurate identification of other PTMs, including those that are particularly rare.

Despite these challenges, our proof of principle experiment is the first example of profiling cysteine thiol oxidation, phosphorylation, and acetylation in β-cells. To the best of our knowledge, it also conferred the highest coverage of multiple PTMs with less fractions according to unique site-level identifications (25, 34, 43, 45). As a result, a number of discoveries were unveiled: new PTM sites, inflammatory biomarkers containing multiple PTMs, and temporal regulatory interactions that appear to be a function of PTM type along with protein abundance. Some combinatorially modified biomarkers include but are not limited to PDX1 (S269, S273, K109, etc.), STAT1 (C155, S727, K379, etc.), PARP14 (C1563, S1407, K26, etc.), and interferon-inducible GTPases (*e.g.*, GBP7 at C12, S591, K361, etc.). Using the PhosphoSitePlus.org mouse databases (137), we identified 9479 putatively new phosphosites and 5276 putatively new acetylation sites (Supplemental Table S11). Because our method is expandable and does not use tags like CysPAT that invariably affect the sample splitting and enrichment steps, we expect that our method can profile additional PTMs and endeavor to implement ubiquitylome enrichment in the near future (24, 25). We are also developing an automated method for RAC enrichment of cysteine-containing peptides, which will fully automate the multi-PTM workflow and help adapt it for data-independent acquisition–based approaches (141). The rich datasets generated using our platform will indulge advancing bioinformatics approaches that incorporate machine learning to interpret multi-PTM interplay (at the intra-protein, inter-protein, and pathway levels) and provide predictions for the role of aberrant PTMs in diseases (142, 143, 144).

## Data Availability

All proteomics data generated for this study have been deposited in the MassIVE repository. The dataset can be accessed using the identifier MSV000095264 after entering the username (MSV000095264) and password (PTM5759).

## Supplemental data

This article contains supplemental data.

## Conflicts of Interest

The authors declare no competing interests.
