# Supplementary material for: Integrative Multi-PTM Proteomics Reveals Dynamic Global, Redox, Phosphorylation, and Acetylation Regulation in Cytokine-Treated Pancreatic Beta Cells
Source: Mol Cell Proteomics. 2024 Nov 15;23(12):100881. doi: 10.1016/j.mcpro.2024.100881 (PMC11700301; doi:10.1016/j.mcpro.2024.100881)
Supplement: Supplemental Material [file mmc12.docx]

**Supporting Information:**

Integrative Multi-PTM Proteomics Reveals Dynamic Global, Redox, Phosphorylation, and Acetylation Regulation in Cytokine-treated Pancreatic Beta Cells

**Authors:**

Austin Gluth ^a,b^, Xiaolu Li ^a^, Marina A. Gritsenko ^a^, Matthew J. Gaffrey ^a^, Doo Nam Kim ^a^, Priscila M. Lalli ^a^, Rosalie K. Chu ^a^, Nicholas J. Day ^a^, Tyler J. Sagendorf ^a^, Matthew E. Monroe ^a^, Song Feng ^a^, Tao Liu ^a^, Bin Yang ^b^, Wei-Jun Qian ^a^, Tong Zhang ^a,*^

1. Biological Sciences Division, Pacific Northwest National Laboratory, Richland, WA, 99352, USA
2. Department of Biological Systems Engineering, Washington State University, Richland, WA, 99354, USA

* Corresponding Author

Dr. Tong Zhang

Biological Sciences Division

Pacific Northwest National Laboratory

Richland, WA 99352

**Tel:** (509)371-7780

**Email:** [tong.zhang@pnnl.gov](mailto:tong.zhang@pnnl.gov)

**Contents:**

(A) Supplementary Figures (pg. S3–21)

(B) Methods (pg. S22–25)

(C) References (pg. S26)

**A. Supplementary Figures**


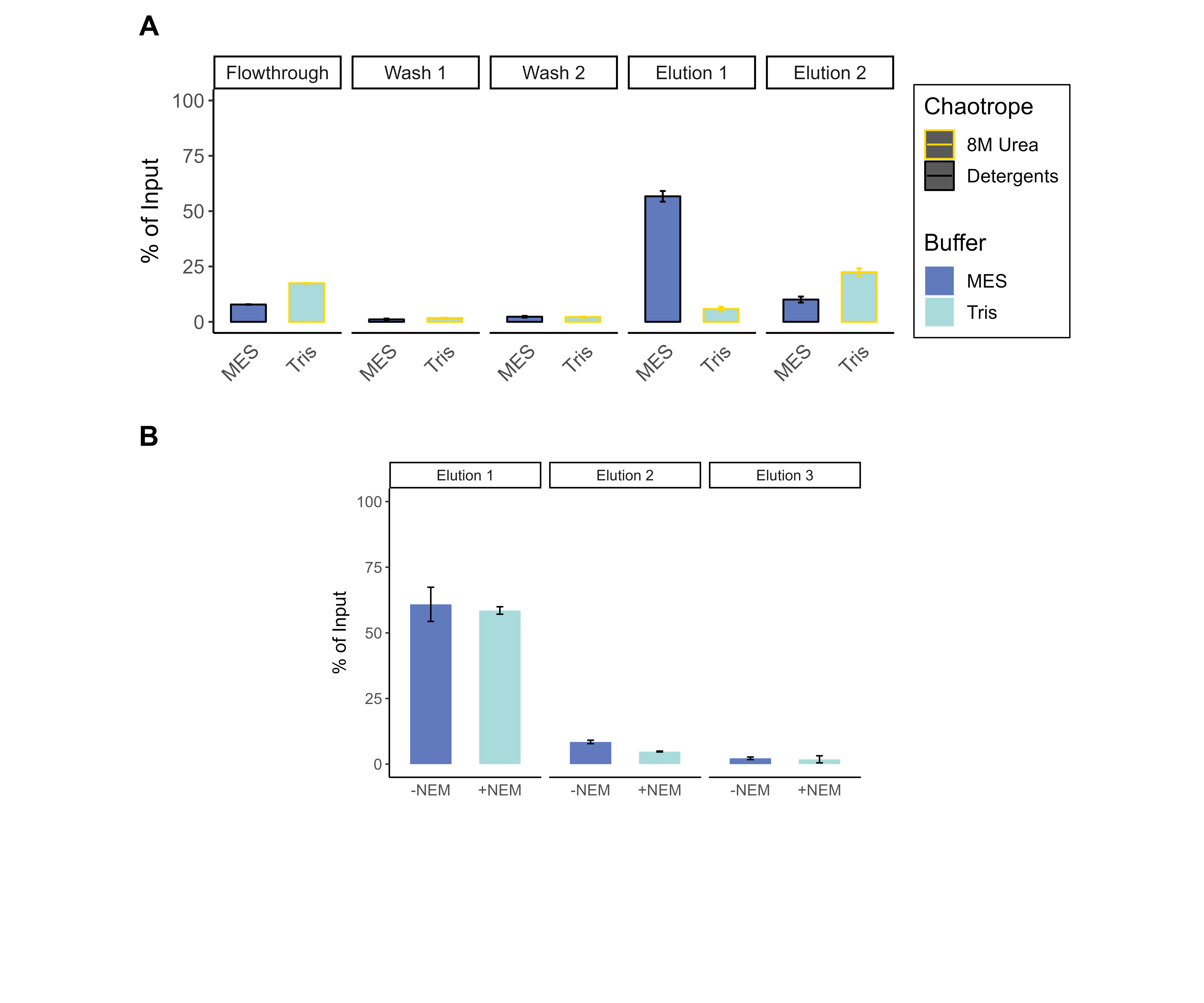
**Figure S1.** Evaluation of MES and NEM compatibility with SP3. ***A,*** Bar chart detailing the BCA analysis of supernatants collected during different stages of SP3. Testing was conducted with BSA. The “MES” buffer system was comprised of 250 mM MES (pH 6.0), 1% SDS, and 1% Triton X-100 and was compared to a published “Tris” buffer system comprised of 100mM Tris (pH 8.0), 8 M urea, and 150 mM NaCl. Here, 250 mM HEPES (pH 7.0) with 2 M urea was used for the elution steps. Error bars are standard deviations of duplicates. ***B,*** Bar chart comparing the MES buffer system with and without 100 mM NEM. Here, 50 mM TEAB (pH 8.0) + 1:50 Trypsin:Protein was used for “Elution 1”, whereas buffer alone was employed for subsequent elutions. Error bars are standard deviations of duplicates.


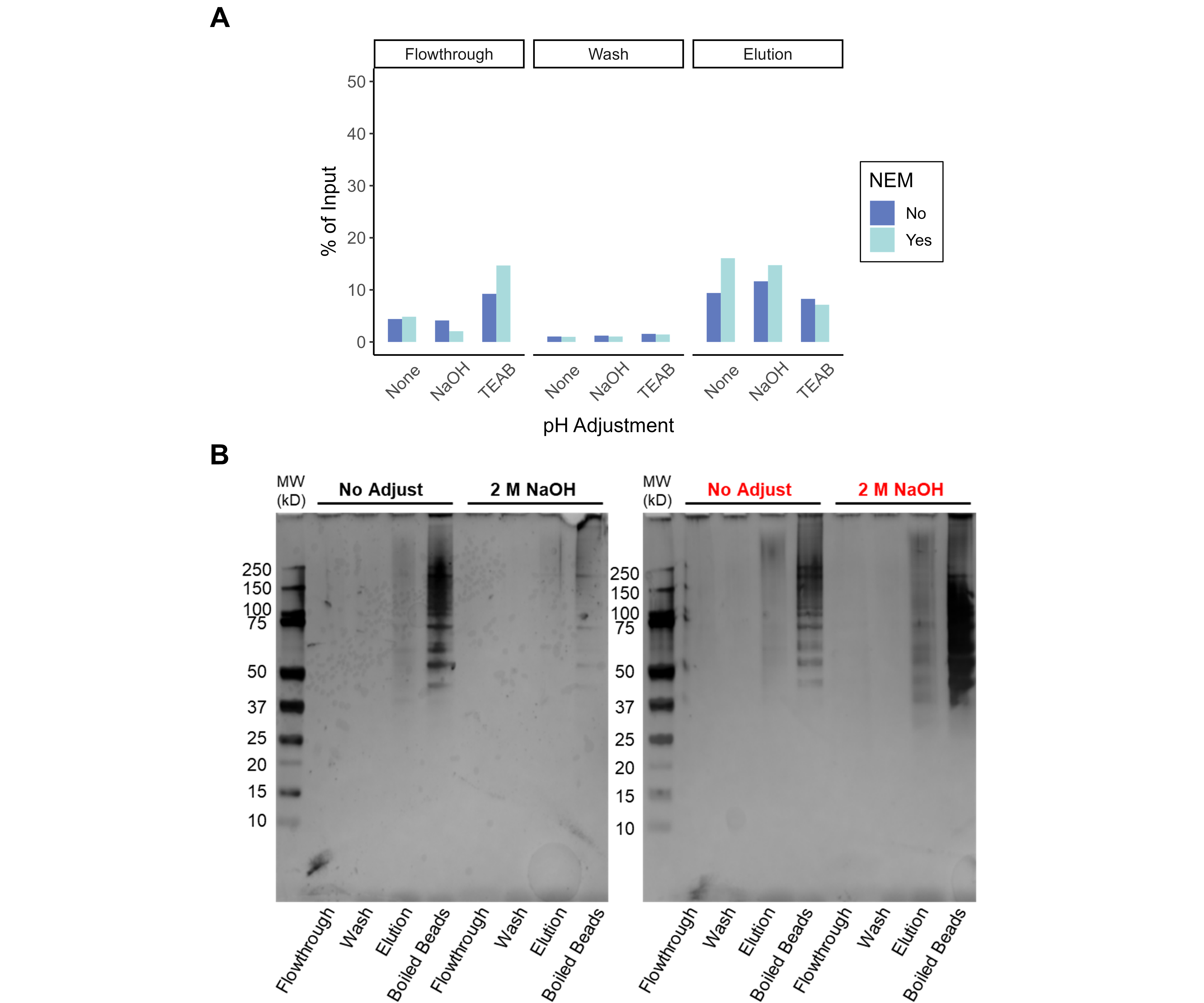
**Figure S2.** Testing pH adjustment prior to the SP3 protein binding step. ***A,*** Bar chart showing the BCA analysis of supernatants collected during different stages of SP3. On the x-axis, “None” means no pH adjustment, “NaOH” corresponds to the use of 2 M NaOH to raise the pH of the MES buffer system to ~8.0, and “TEAB” corresponds to using 1 M TEAB (pH 8.5) to raise the pH to ~8.0. Only supernatants from the first wash (“Wash”) were analyzed. Singlets were evaluated for this experiment. ***B,*** Images of silver-stained SDS-PAGE gels demonstrating protein binding during SP3. The gel on the left included –NEM samples, while the gel on the right (red text) included +NEM samples. Data for fractions from the TEAB condition not shown.


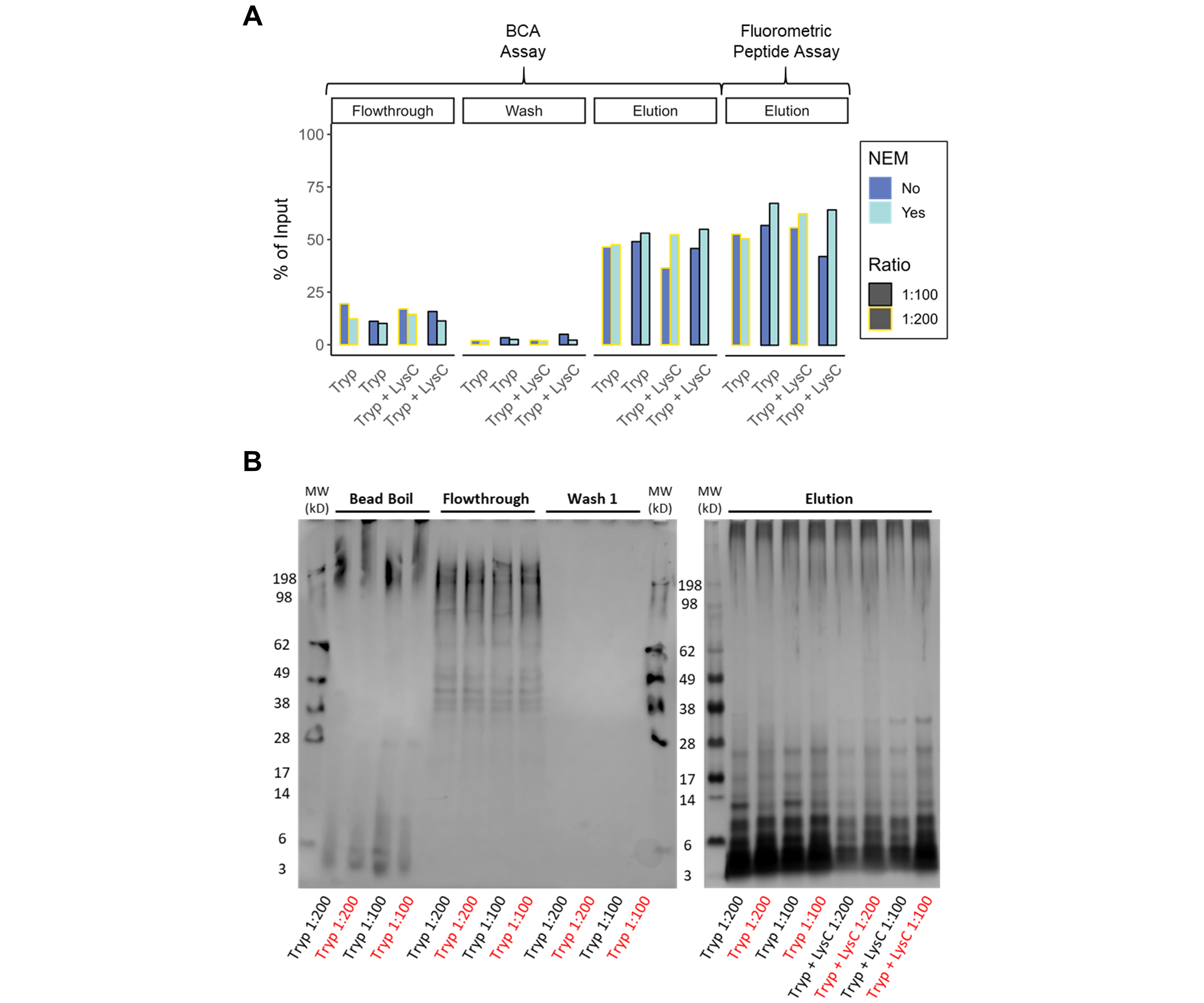
**Figure S3.** Confirming peptide elution from SP3 using low protease ratios. ***A,*** Bar chart showing the BCA analysis of supernatants collected during different stages of SP3. Only supernatants from the first wash (“Wash”) were analyzed. Singlets were evaluated for this experiment. The Pierce Quantitative Fluorometric Peptide Assay was also used to evaluate digest elution. “Tryp” corresponds to “Trypsin”. ***B,*** Images of silver-stained SDS-PAGE gels demonstrating protein binding (left; equal volumes loaded) and peptide elution (right; 10 μg loaded) during SP3. Red labels represent samples blocked with NEM. SeeBlue Plus2 (Invitrogen) ladder included.


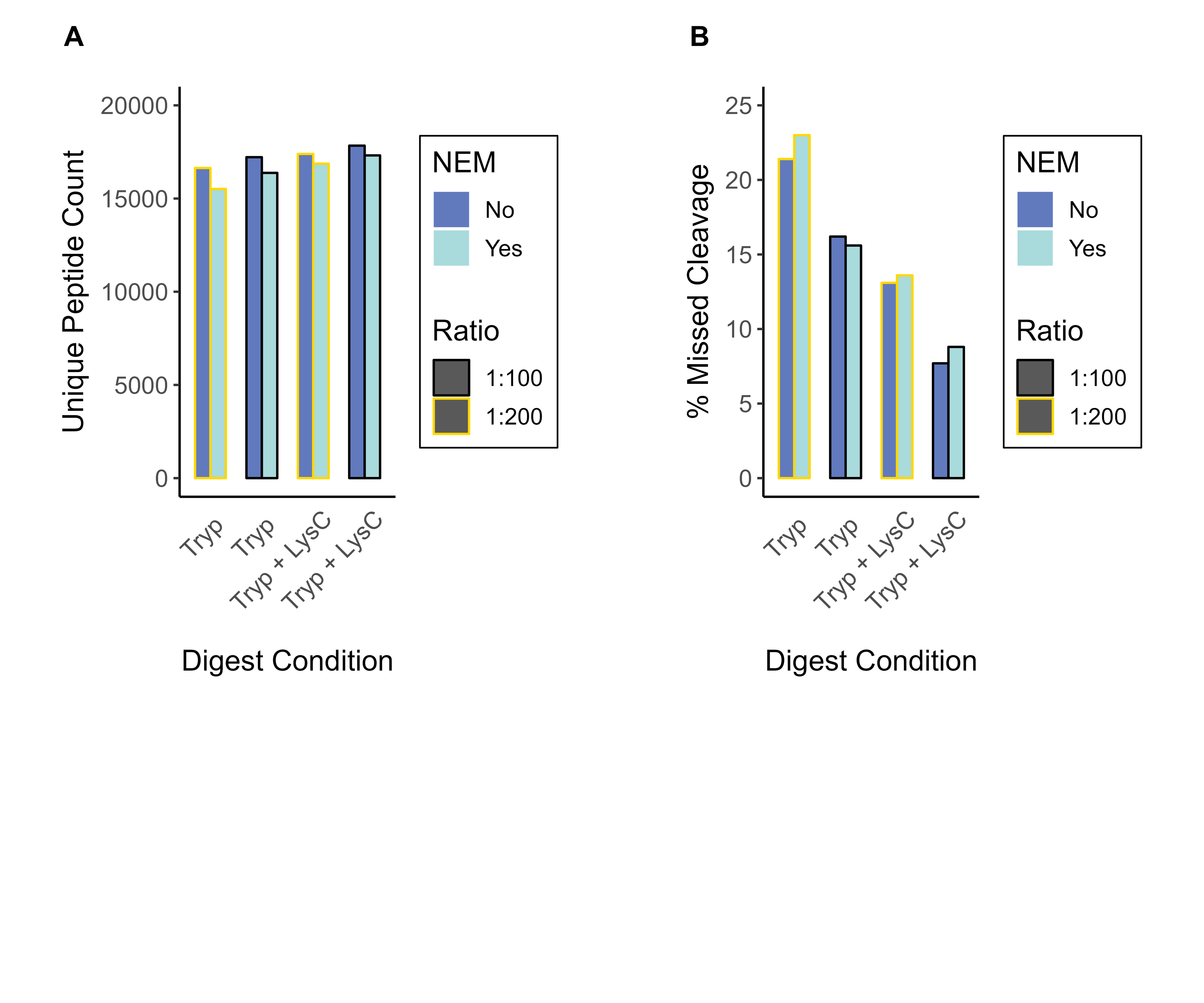
**Figure S4.** Single-shot LC-MS/MS analysis of on-bead digests using low protease ratios. ***A,*** Bar chart of unique peptide identifications for the Trypsin (“Tryp”) vs. Trypsin + LysC digest conditions. Singlets were evaluated for this experiment. ***B,*** Bar chart showing % missed cleavage for singlets from two digest conditions.


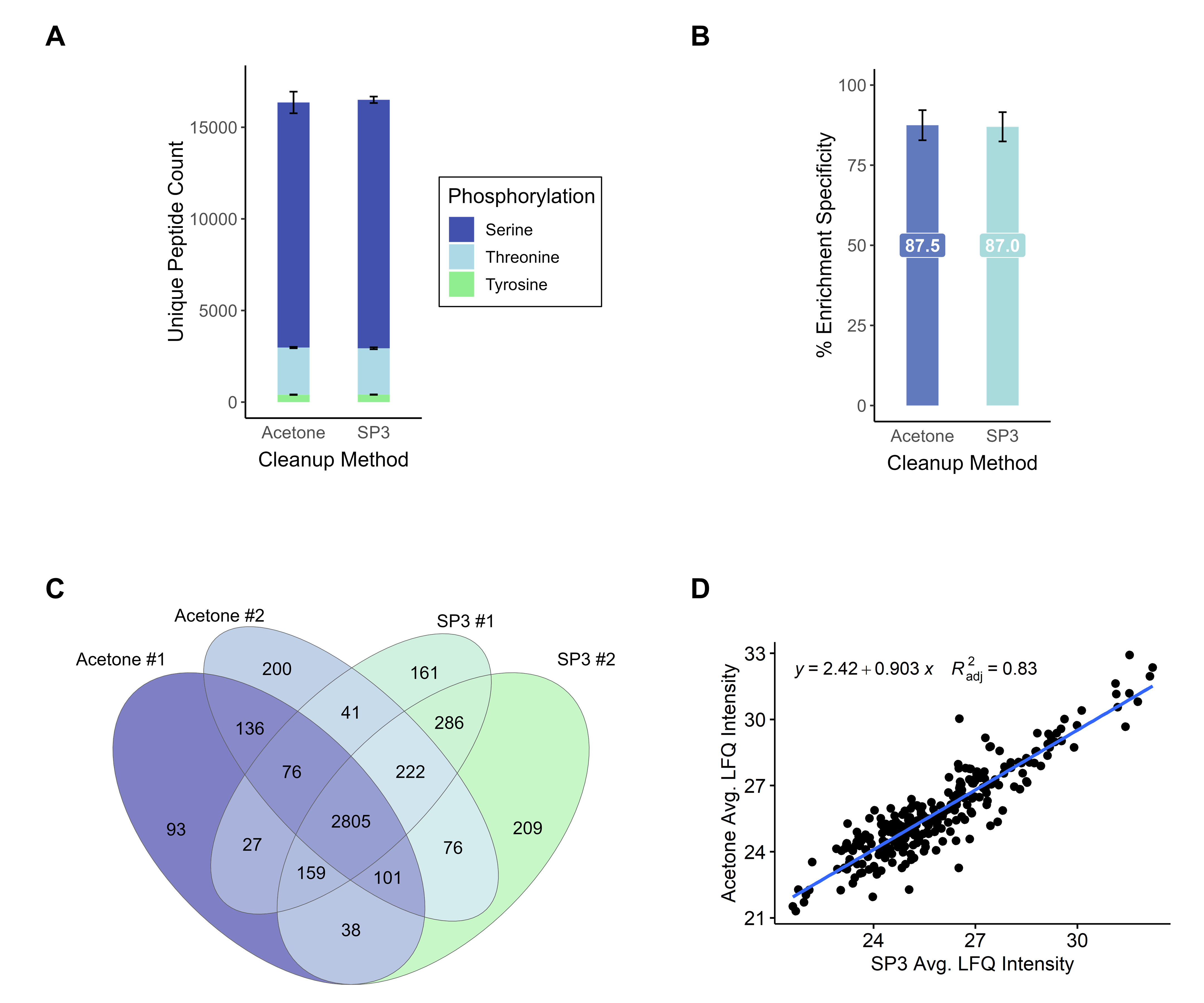


**Figure S5.** Compatibility of NEM-blocked samples with phosphopeptide enrichment. ***A****,* Stacked bar chart comparing the phosphopeptide coverage and phosphorylated residue profile attained by acetone precipitation vs. SP3. Error bars are standard deviations of duplicates. ***B****,* Bar chart showing the phosphopeptide enrichment selectivities for acetone precipitation vs. SP3. ***C****,* Venn diagram presenting protein coverage overlap for duplicate samples processed by acetone precipitation vs. SP3. ***D***, Scatter plot of average LFQ intensities (duplicates) for overlapping protein identifications from acetone precipitation and SP3. The equation for linear best-fit and the corresponding adjusted R^2^ value are included in the plotting space.


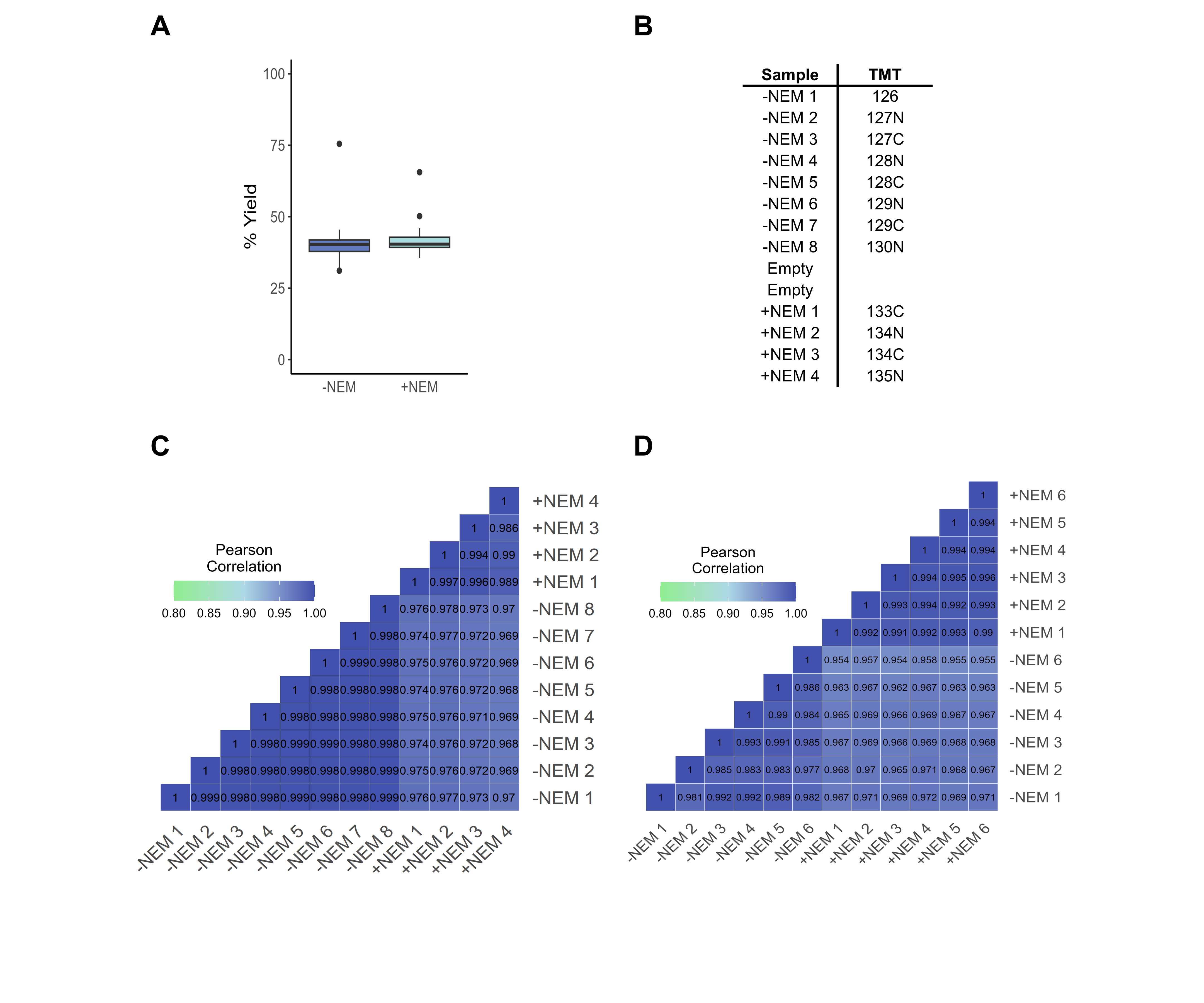


**Figure S6.** Evaluating the automated SP3 workflow to process 200 μg inputs. ***A,*** Box plot showing peptide yields after elution using automated SP3. Six replicates of samples with and without NEM were analyzed. “% Yield” designates the percentage of the input attained after SP3. ***B,*** TMT labeling scheme used for this 200 μg input experiment. ***C,*** Correlogram of multiplexed quantification results from TMT-labeled global samples following automated SP3 using 200 μg protein inputs. ***D,*** Correlogram of label-free quantification results for global samples derived from automated SP3 workflow.


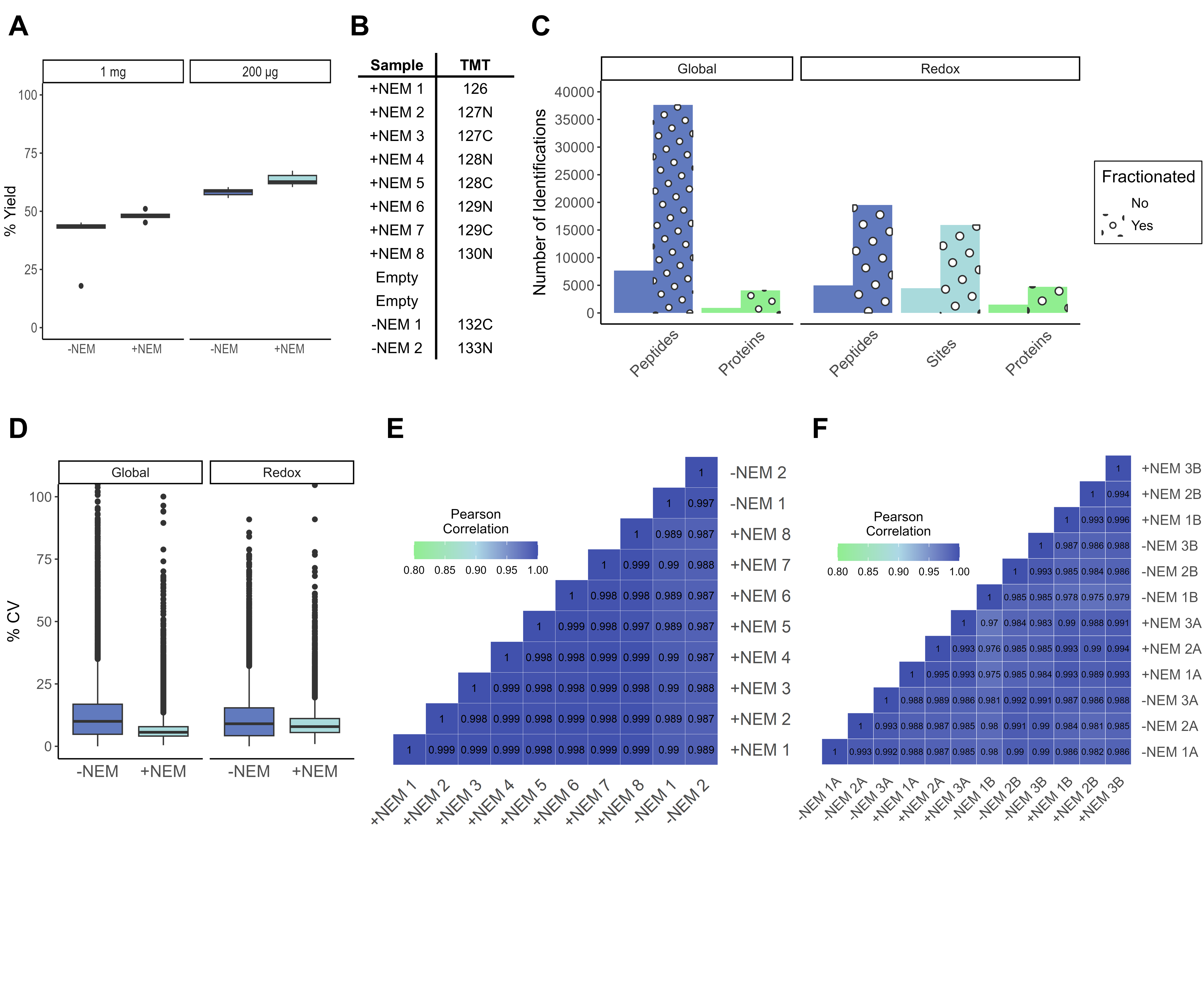
**Figure S7.** Deep global and redox profiling using the automated SP3 workflow to process 1 mg inputs. ***A,*** Box plot showing peptide yields following the digest/elution step of the optimized automated SP3 method (see Results and Methods sections for details). Six replicates of samples with and without NEM were analyzed for the two protein inputs tested. ***B,*** TMT labeling scheme used for this 1 mg input experiment. ***C,*** Bar chart demonstrating the effect of fractionation on the number of unique identifications. ***D,*** Box plots showing the distribution of peptide level CV following median normalization. Note that the number of replicates varies for Oxidation (+NEM) and Total Thiol (−NEM) samples as shown in panel B*.* ***E,*** Correlogram of multiplexed quantification results from TMT-labeled global samples following automated SP3. Digested peptides from the 1 mg protein input condition were used for labeling. ***F,*** Correlogram of label-free quantification results for global samples derived from the automated SP3. 200 μg protein inputs (series “A”) were compared with 1 mg inputs (series “B”) in the same experiment.


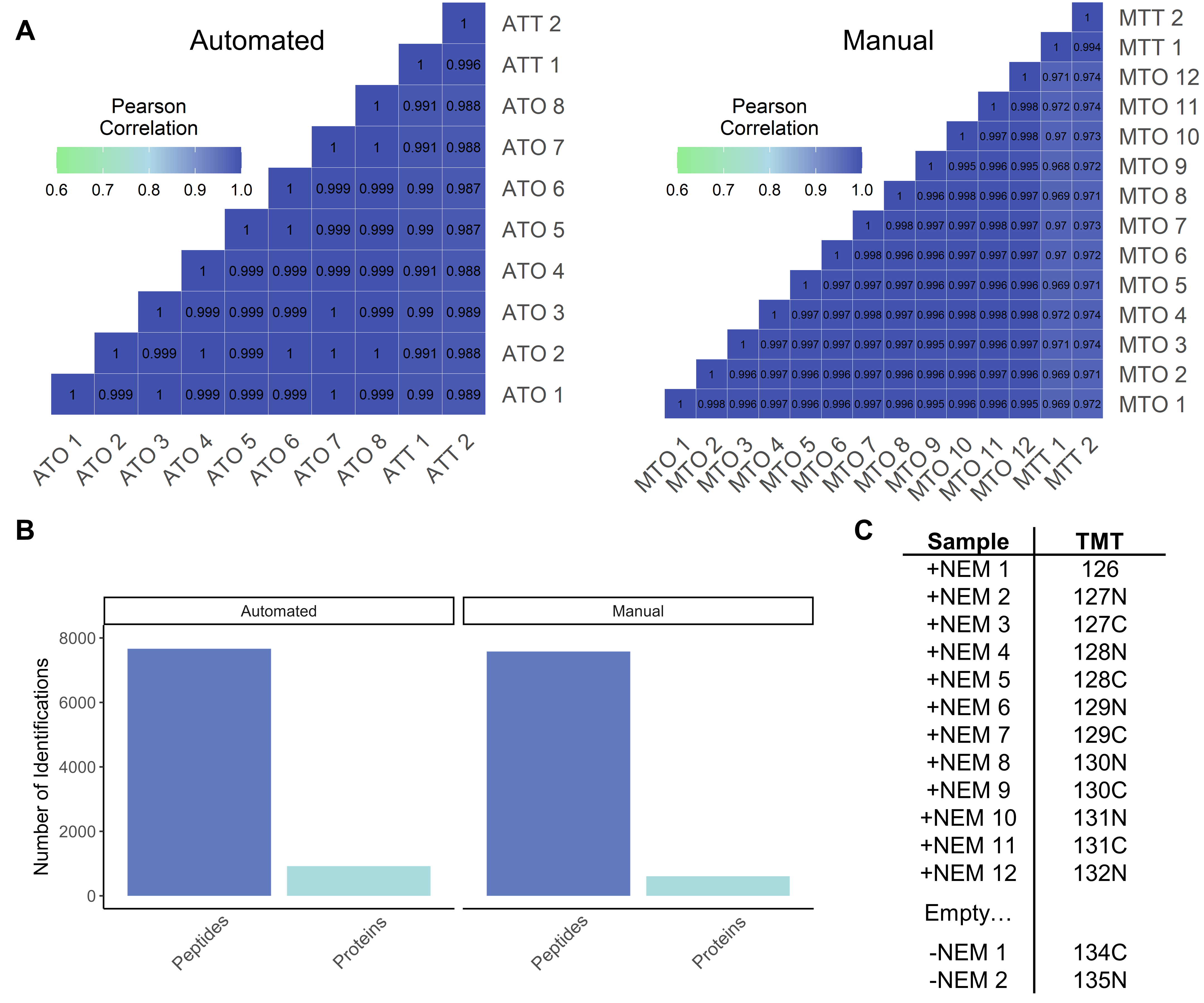
**Figure S8.** Comparing the automated and manual SP3 workflows using 1 mg protein inputs. ***A,*** Correlograms of multiplexed quantification results for global samples derived from the automated (“A”) vs. manual (“M”) SP3 workflows that used the same skeletal tissue lysate inputs. Here, “TO” corresponds to Thiol Oxidation (+NEM), while “TT” stands for Total Thiol (−NEM). ***B,*** Bar chart showing the unique peptide and protein identifications from the global TMT-labeled samples for both workflows. The results from automated SP3 are the same as the unfractionated results shown in supplemental **Fig. S7C**. ***C,*** The TMT labeling scheme used for the manual workflow in this comparison. Note that the number of replicates varies for Thiol Oxidation (+NEM) samples. Refer to supplemental **Fig. S7B** for the labeling scheme used for the automated SP3 experiment.


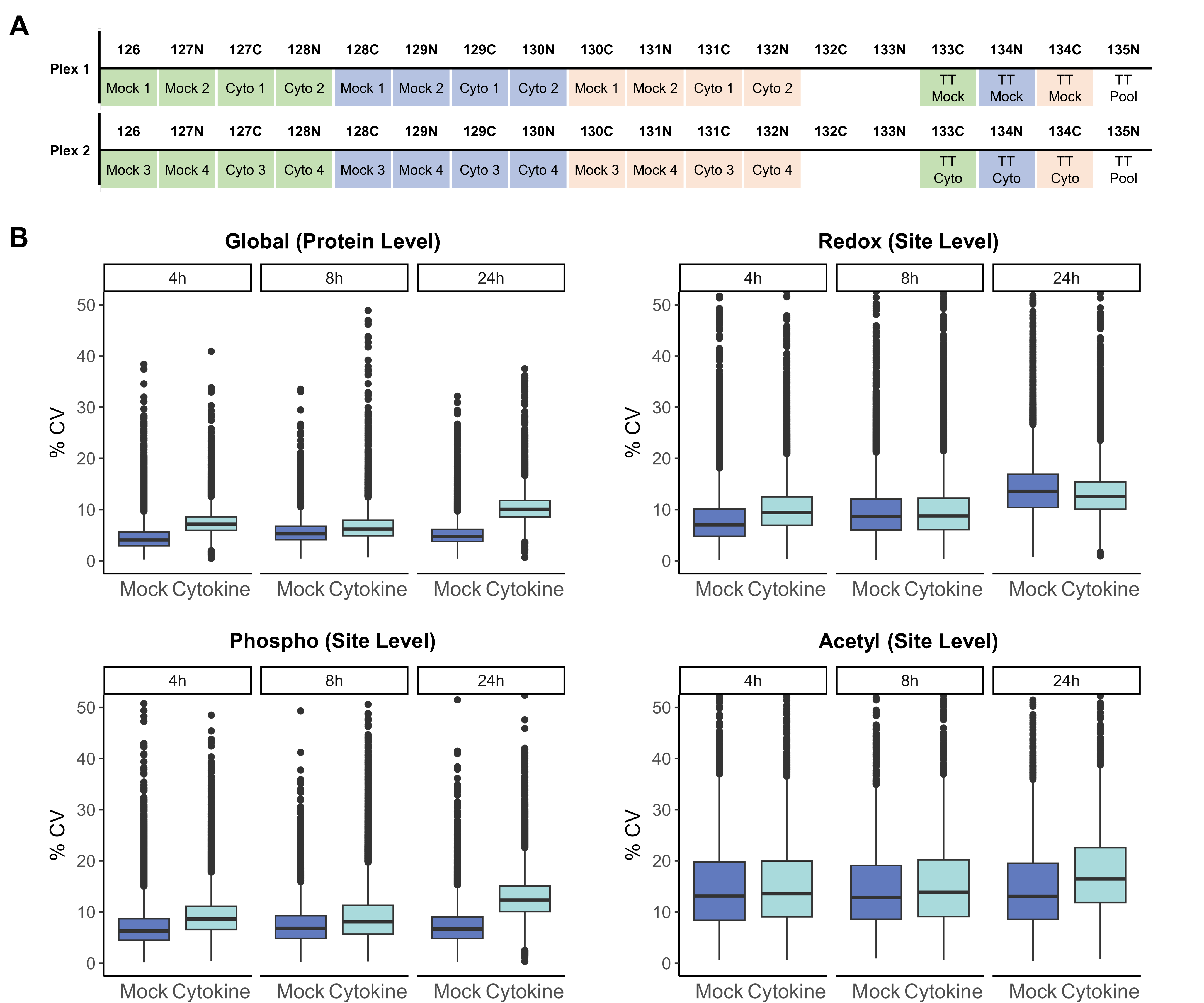


**Figure S9.** Evaluating the variability of Thiol Oxidation (+NEM) replicates from the multiplexed β-cell timecourse experiment. ***A,*** The multiplex TMT labeling scheme. Biological quadruplicates of mock (“Mock”) and cytokine-treated (“Cyto”) samples were split between two plexes. Green indicates 4 h samples, blue indicates 8 h samples, and orange indicates 24 h samples. “TT” refers to the “Total Thiol” channels, and “TT Pool” designates the channels in which total thiol peptides were pooled in equal quantities. ***B,*** Box plots showing the distribution of protein level and site level CVs after batch correction. “Mock” refers to the untreated control.


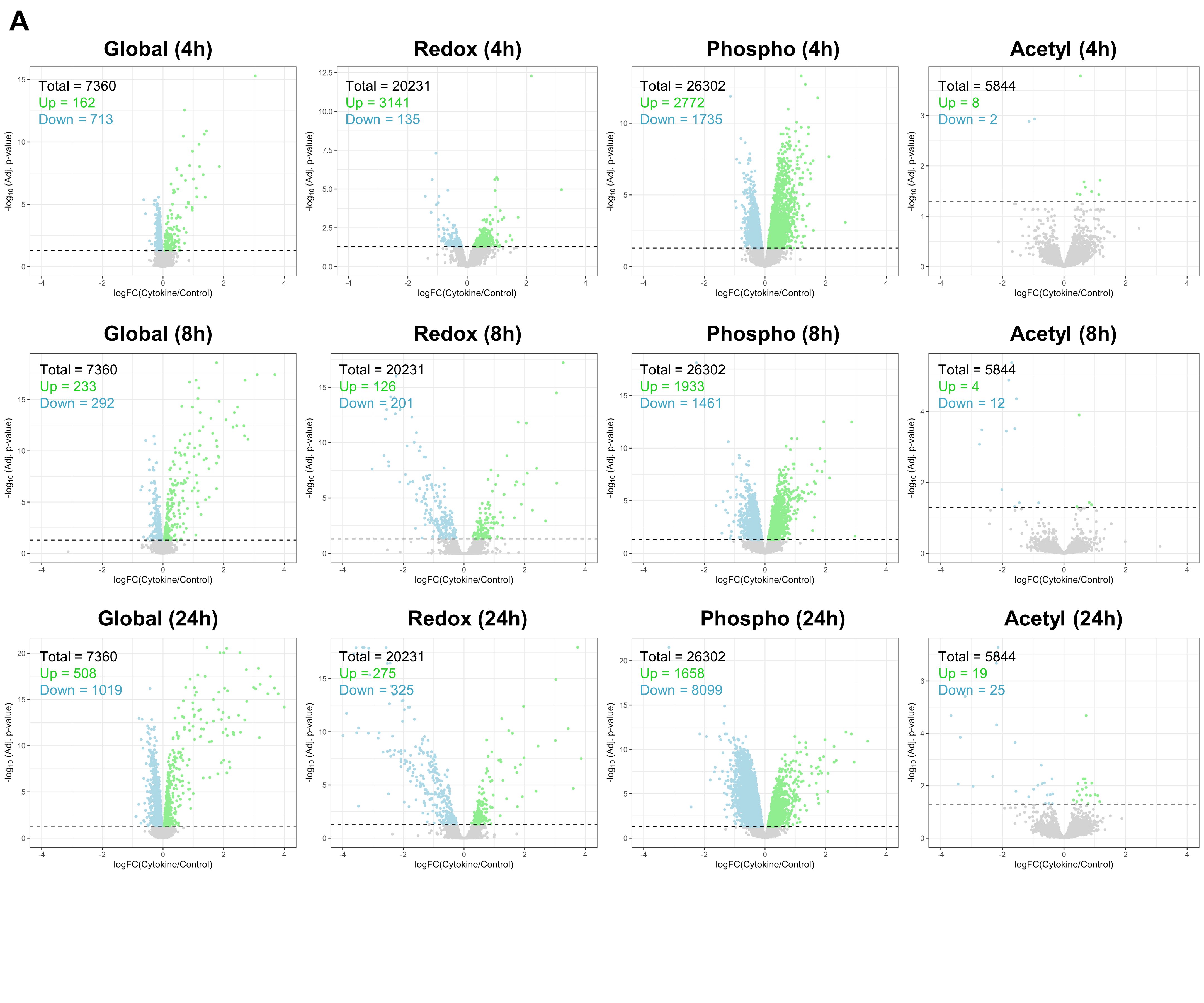
**Figure S10.** Volcano plots showing relative abundance changes during the β-cell timecourse experiment. ***A,*** The volcano plots contain –log_10_ limma adjusted p-values on the y-axis and log_2_ transformed fold changes (Cytokine/Control) on the x-axis. The dotted line in each plot specifies an adjusted p-value of 0.05. The “Total” number of unique protein and site IDs are included in each plot along with the number of IDs that passed the adjusted p-value threshold and were upregulated (“Up”) or downregulated (“Down”).


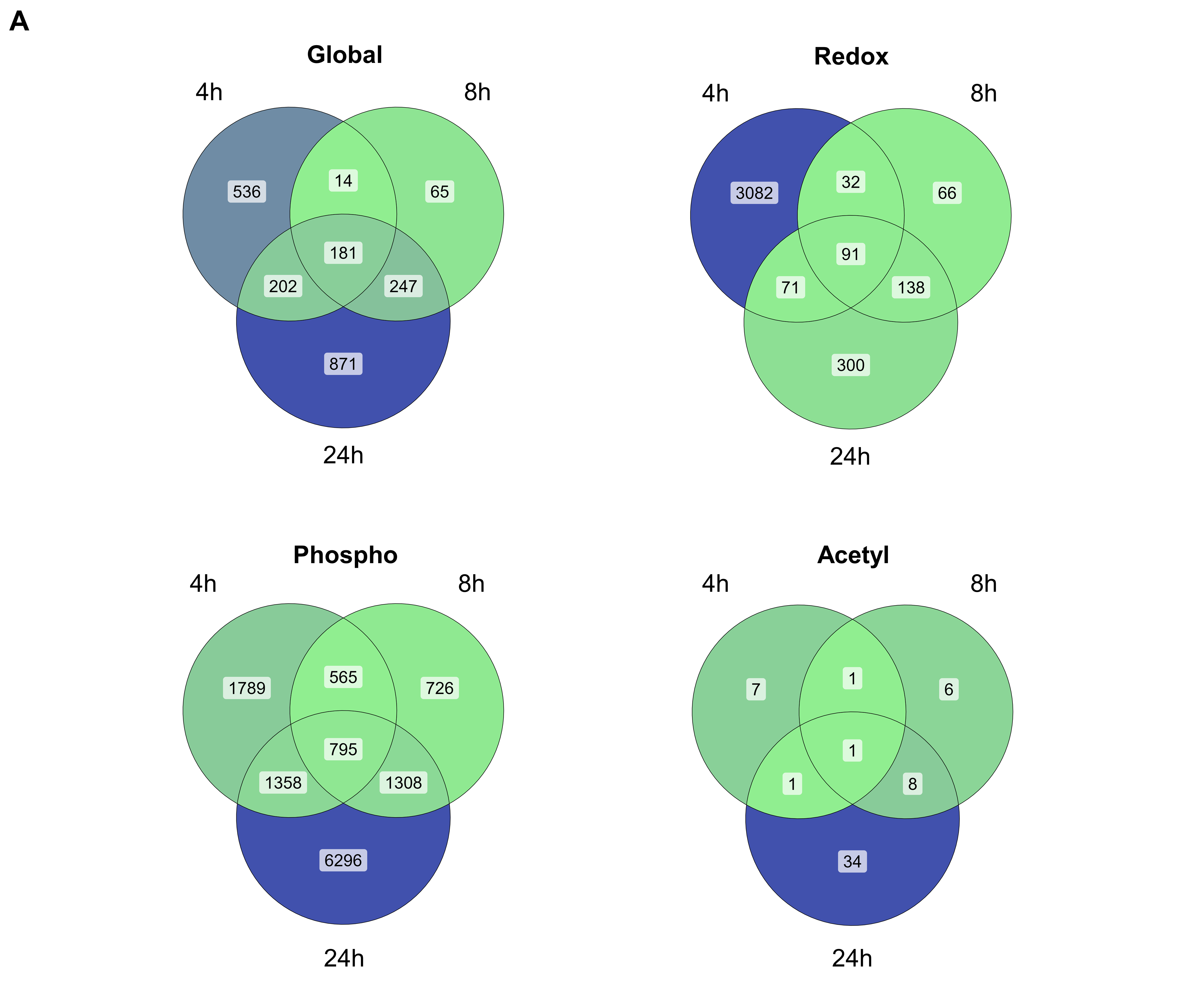
**Figure S11.** Venn diagrams depicting the overlap of unique IDs from differential expression analyses of the β-cell timecourse experiment. ***A,*** Unique IDs at the protein level and site level are derived from pairwise two-sample t-tests between the cytokine condition and the time-matched control. The displayed counts are for IDs that passed an adjusted p-value of 0.05.


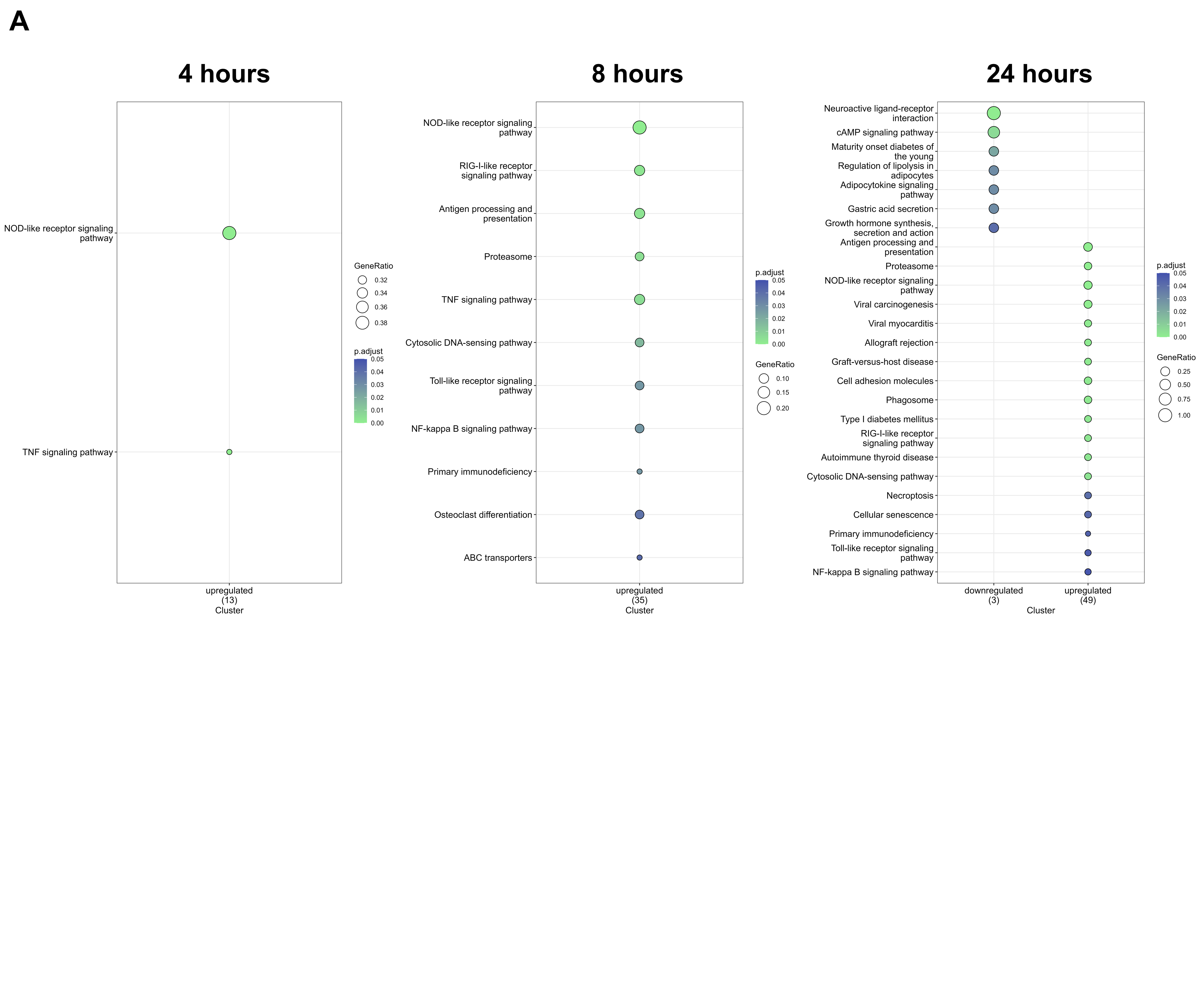
**Figure S12.** Dot plots showing results of KEGG pathway over-representation analyses for global proteomics samples from the β-cell timecourse experiment. ***A,*** KEGG ORA pathway enrichment analyses for were conducted using IDs from differential expression analyses that had an absolute log_2_FC ≥ 0.8 and an adjusted p-value ≤ 0.05. Enrichment results were filtered using an adjusted p-value cutoff of 0.05 and a q-value cutoff of 0.1. Subcategories for “Infectious diseases” and “Substance dependence” were removed prior to visualization.


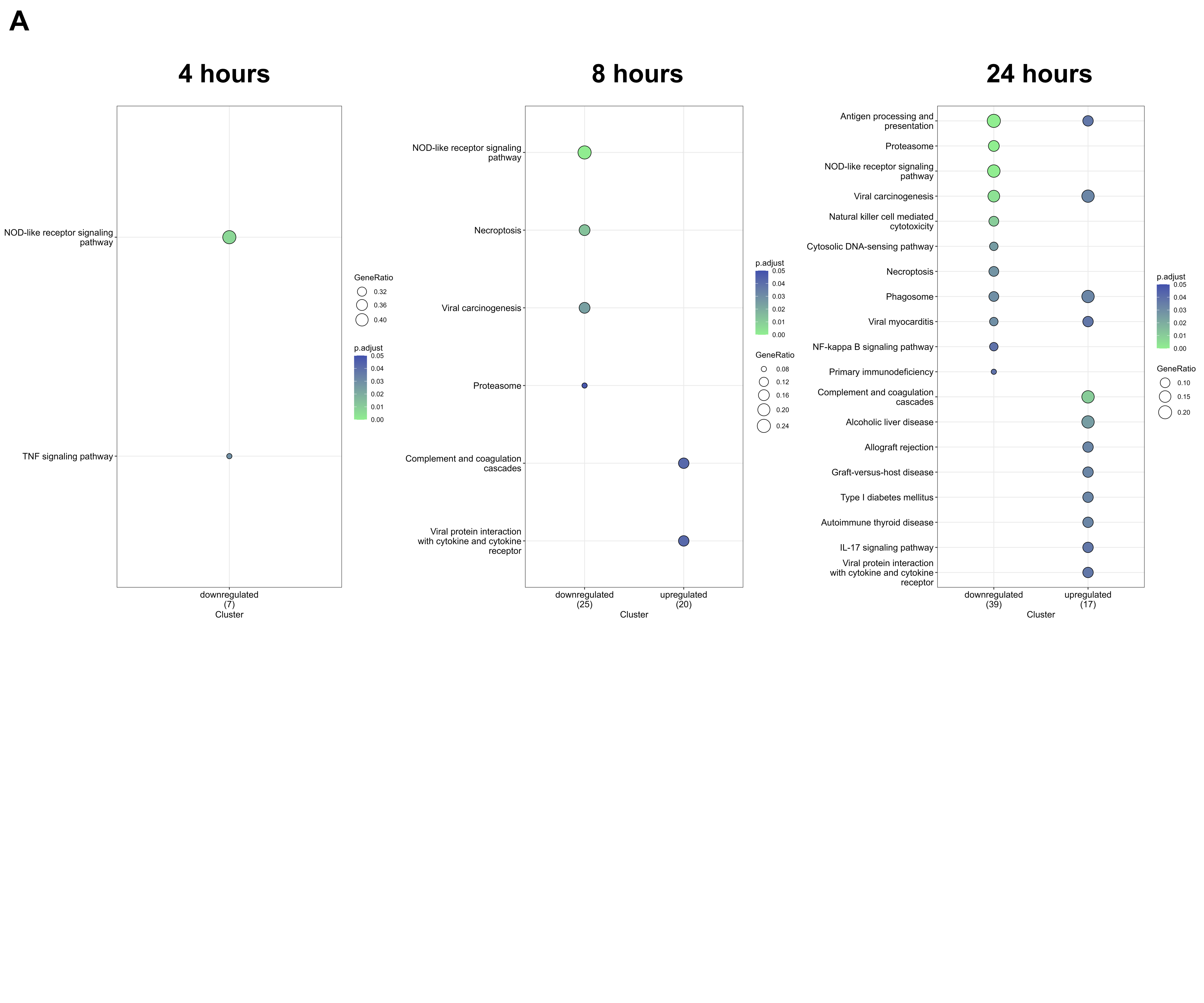
**Figure S13.** Dot plots showing results of KEGG pathway over-representation analyses for redox proteomics samples from the β-cell timecourse experiment. ***A,*** KEGG ORA pathway enrichment analyses for were conducted using IDs from differential expression analyses that had an absolute log_2_FC ≥ 0.8 and an adjusted p-value ≤ 0.05. Enrichment results were filtered using an adjusted p-value cutoff of 0.05 and a q-value cutoff of 0.1. Subcategories for “Infectious diseases” and “Substance dependence” were removed prior to visualization.


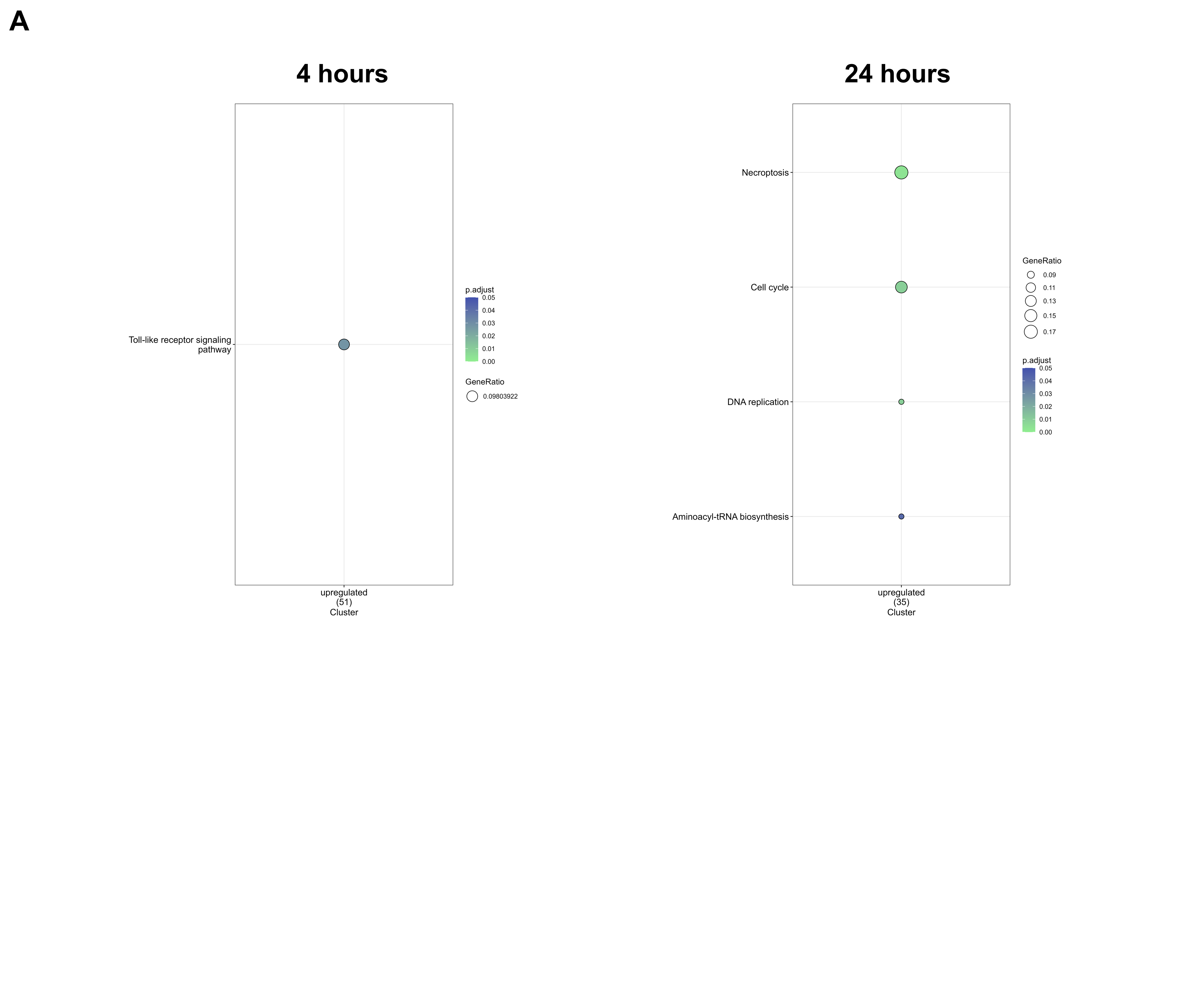
**Figure S14.** Dot plots showing results of KEGG pathway over-representation analyses for phosphoproteomics samples from the β-cell timecourse experiment. ***A,*** KEGG ORA pathway enrichment analyses for were conducted using IDs from differential expression analyses that had an absolute log_2_FC ≥ 0.8 and an adjusted p-value ≤ 0.05. Enrichment results were filtered using an adjusted p-value cutoff of 0.05 and a q-value cutoff of 0.1. Subcategories for “Infectious diseases” and “Substance dependence” were removed prior to visualization. Note that no pathways were over-represented for the 8 hour time point.


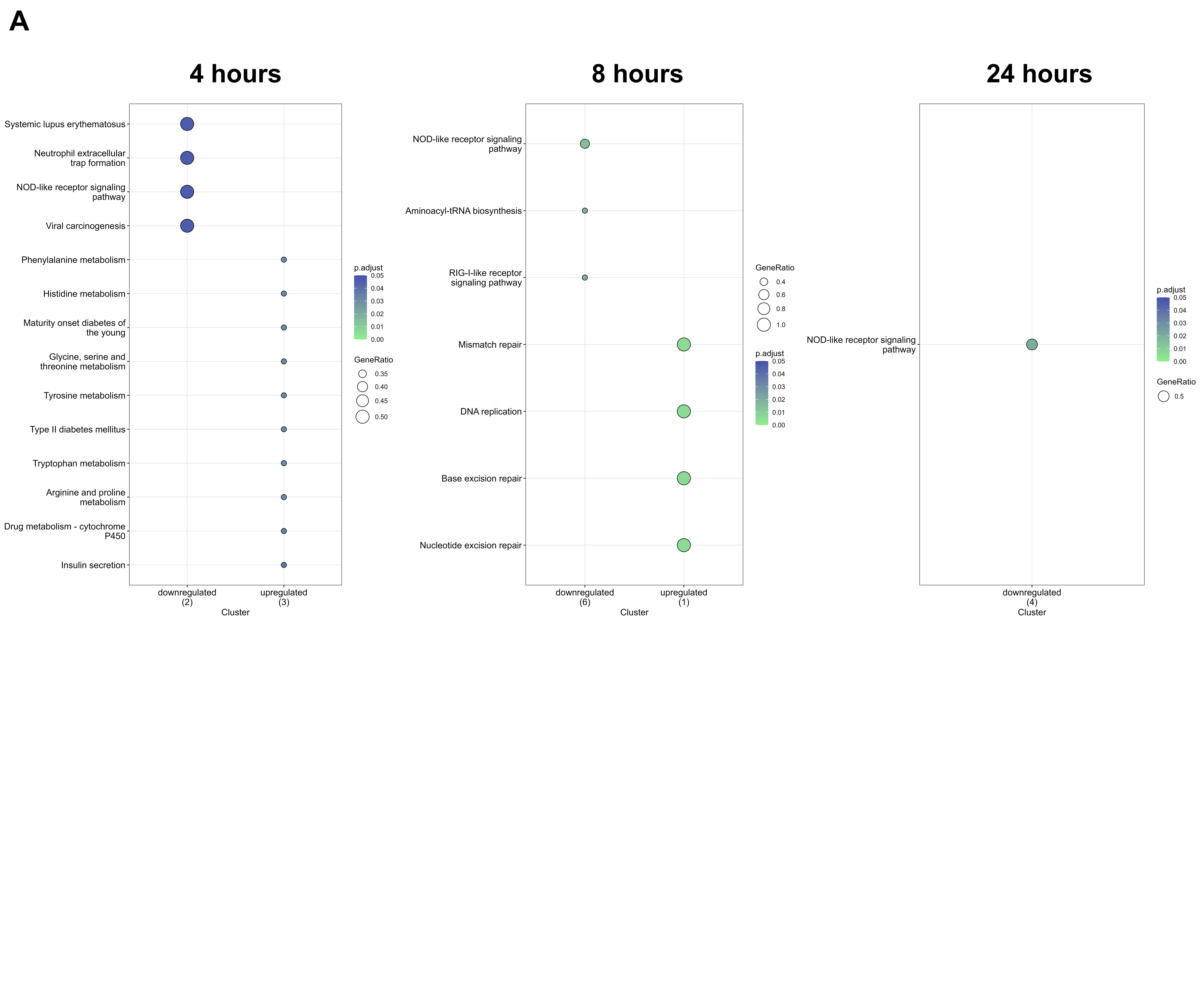
**Figure S15.** Dot plots showing results of KEGG pathway over-representation analyses for acetylomics samples from the β-cell timecourse experiment. ***A,*** KEGG ORA pathway enrichment analyses for were conducted using IDs from differential expression analyses that had an absolute log_2_FC ≥ 0.8 and an adjusted p-value ≤ 0.05. Enrichment results were filtered using an adjusted p-value cutoff of 0.05 and a q-value cutoff of 0.1. Subcategories for “Infectious diseases” and “Substance dependence” were removed prior to visualization.


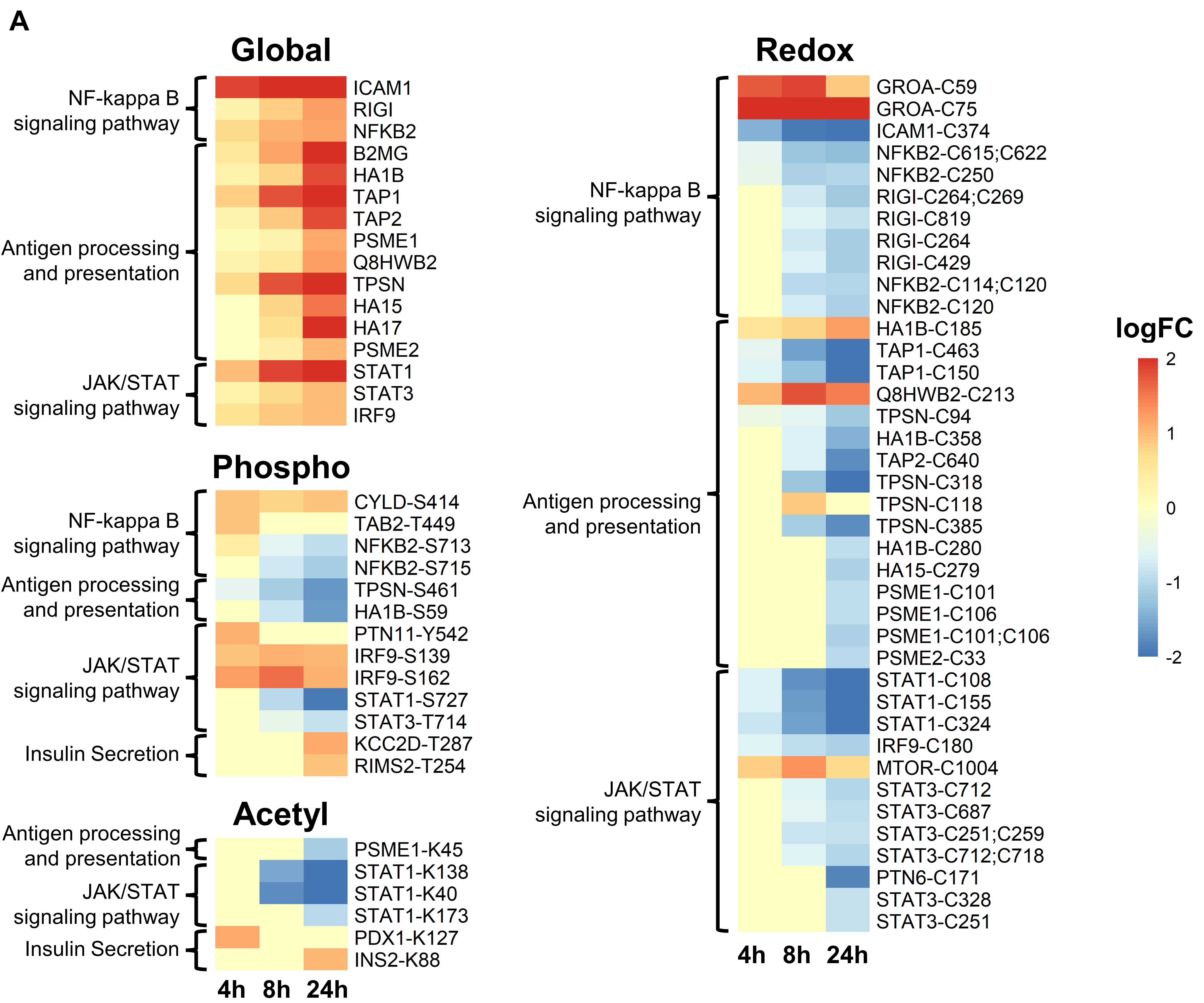
**Figure S16.** Heatmaps showing log_2_FCs over time for select signaling pathways and biological processes. ***A***, These particular pathways were chosen based on KEGG ORA, and proteins/sites with log_2_FCs ≥ 0.8 and adjusted p-values ≤ 0.05 are shown. Note some proteins have a substantial increase in relative abundance with a corresponding decrease in cysteine thiol oxidation, which may in some cases be simply described due to the requirement of certain reduced cysteine thiols for protein production (as opposed to those, for example, involved in disulfide bond formation).


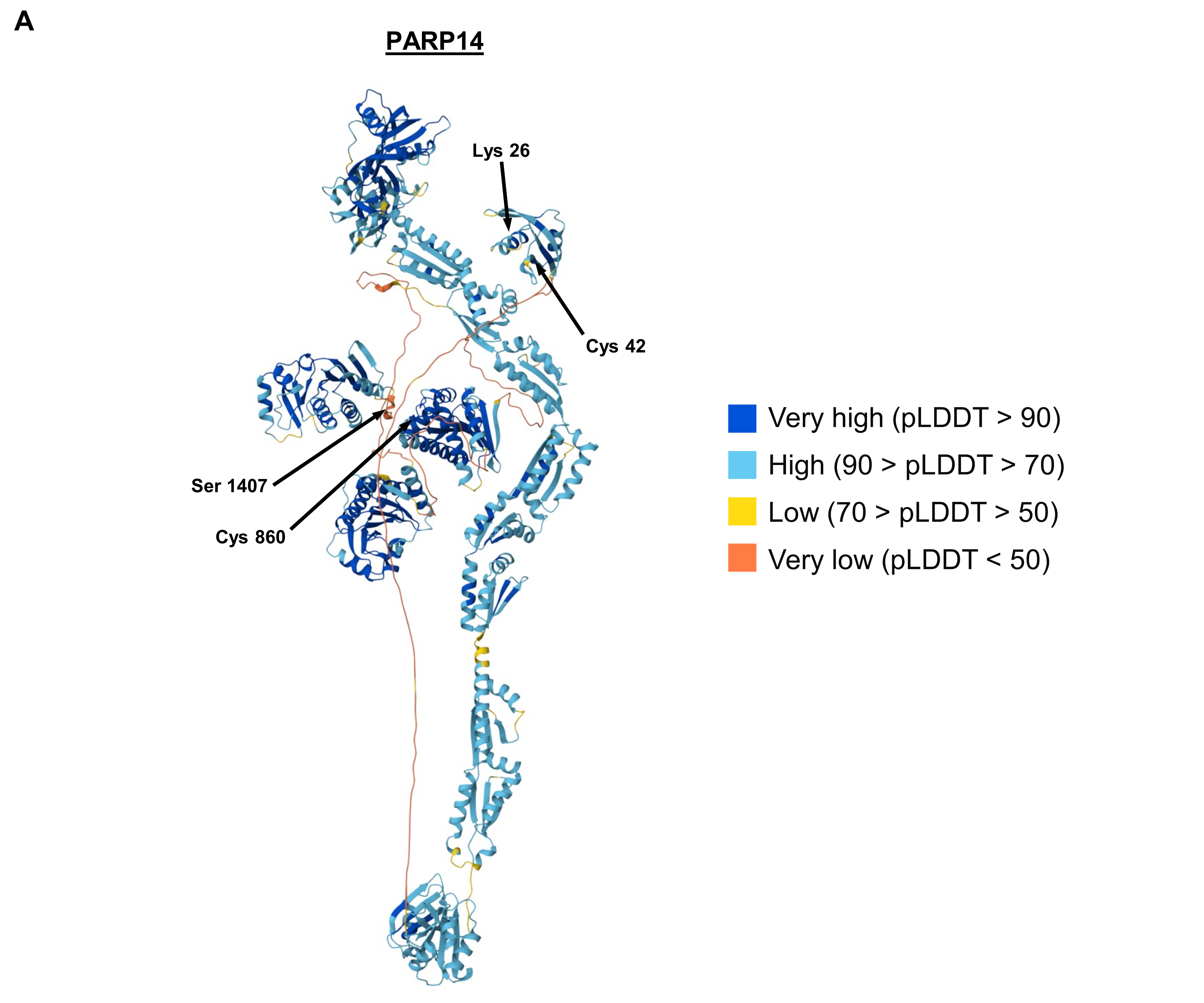
**Figure S17.** Multiple PTMs with differential abundances co-localize in disordered regions. ***A***, AlphaFold-predicted PARP14 structure with overlaid model confidence scores (1). The locations of the residues discussed in the main text are specified. Note the two loops with very low prediction scores: the one on the right contains the phosphorylated S1407 residue.

**B. Methods**

**SP3 Bead Pre-washing.** Beads were removed from storage at 4 °C and equilibrated to RT for 30 min. Beads were resuspended by gentle mixing, and a 1:1 mix of hydrophobic and hydrophilic beads were made (up to 25 mg in a 1.5 mL microcentrifuge tube). This tube was placed on an Invitrogen DynaMag-2 magnetic stand for 2 min to collect the beads, and the storage buffer was removed. The beads were washed by adding 1 mL of 50 mM HEPES (pH 9.1) containing 200 mM NaCl to remove the storage buffer and disaggregate the beads. The beads were incubated at RT and 850 rpm shaking for 2 min and then placed on the magnetic stand for 2 min to collect the beads and remove the wash buffer. This procedure was repeated twice more followed by additional washes using 1 M NaCl to remove residual bead encapsulation polymer. Finally, beads were washed five times with ultrapure water and resuspended at a final concentration of 50 μg/μL.

**MES Buffer and NEM Compatibility Tests Using BSA.** SP3 bead information and preparation details are provided in the methods section of the main text. BSA (Sigma-Aldrich) was weighed to prepare a fresh 1 mg/mL stock, and 200 µg was transferred to test two buffer systems: 250 mM MES (pH 6.0) + 1% SDS + 1% Triton X-100 vs. 100 mM Tris (pH 8.0) + 8 M urea + 150 mM NaCl, which was used by Leutert et al. (2). The pH of the MES buffer system was adjusted to 8.0 using 2 M NaOH. Following pre-washing, SP3 beads resuspended at a final concentration of 50 μg/μL were added to the BSA samples at a 10:1 bead:protein ratio. The samples were then incubated for 10 min at RT with shaking (1000 rpm), and 400 μL of absolute ethanol was added. The samples were then incubated for 5 min at RT with shaking (1000 rpm), after which they were placed on a magnetic rack for 1 min. The supernatants were removed and designated as “Flowthrough”. The samples were removed from the rack, and 400 μL of 80 vol% ethanol was added. The samples were incubated for 5 min at RT with shaking (1000 rpm) and washed once more in the same manner. The supernatants from each wash were saved and labeled “Wash”. The proteins were eluted by adding 200 μL of 250 mM HEPES (pH 7.0) + 2 M urea and incubating for 5 min at RT with shaking (1000 rpm). A second elution using the same buffer was conducted and analyzed separately (“Elution 2”). Flowthrough and wash fractions were placed in a speedvac to dry and resuspended in 200 μL of 250 mM HEPES (pH 7.0) + 2 M urea. Finally, a yield analysis was conducted using BCA.

For the NEM compatibility tests, 100 mM was tested with the MES buffer system described above: 200 μg of BSA was incubated at 55 °C and 850 rpm shaking for 30 min. for NEM alkylation of free thiols. The SP3 protocol detailed above was used; however, an actual digestion step was used to elute peptides: 50 mM TEAB (pH 8.0) + 1:50 Trypsin:Protein Input for “Elution 1” then 50 mM TEAB (pH 8.0) for subsequent elutions.

**Evaluation of MES Buffer pH.** Cultivation, lysis, and NEM blocking of C10 cells were described in the methods section of the main text. Information about SP3 beads and bead preparation are also detailed in the main text. The MES buffer was either not adjusted (pH 6.0 final) or adjusted to pH 8.0 using 2 M NaOH or 1 M TEAB (pH 8.5). Singlets of –NEM and +NEM samples for each condition were evaluated. 200 µg of protein from C10 lysate was used for each sample with a 200 uL final volume and a bead:protein ratio of 10:1. SP3 was performed as described above; though, only the supernatant from the first wash was analyzed, and two elutions were performed (and then combined) by adding 100 µL of 50 mM TEAB (pH 8.5), incubating at 37 °C for 30 min., and placing samples on the magnetic rack for 2 min. to collect elutions. 200 µL of 1x MES-SDS Running Buffer (Invitrogen) added to the saved SP3 beads followed by vortexing to resuspend. The “Flowthrough” and “Wash” fractions were dried in a speedvac then resuspended in 200 µL of 50 mM TEAB (pH 8.5) via water bath sonication. All fractions analyzed using the BCA assay. 1x Dye and 0.2 M DTT were added to 10 µL aliquots of the various fractions, which were then boiled for 10 min. Finally, NuPAGE 4-12% Tris-Bis gels (Invitrogen) were used for SDS-PAGE with Bio-Rad Precision Plus Protein Standard Ladders. The gels were stained using the Pierce Silver Stain Kit and imaged with Gel Doc EZ Imager (Bio-Rad).

**Simple Evaluation of Digest Efficiency using Modified SP3 Workflow.** Cultivation, lysis, and NEM blocking of C10 cells as well as SP3 cleanup described in the methods section of the main text. Following digestion using different combinations and ratios of Protease:Protein Input, peptides were incubated with 5 mM DTT for 30 min. at 37 °C with 850 rpm shaking. Afterwards, IAM was added to a final concentration of 40 mM, and samples were incubated in the dark at room temperature for 30 min. Formic acid was then added to a final concentration of 0.1%, and samples were desalted using Phenomenex C18E 50mg SPE columns. After drying via speedvac, the samples were resuspended in 5% acetonitrile + 0.1% TFA and transferred to Waters vials at a final concentration of 0.1 µg/µL for LC-MS/MS analysis as described in the main text.

**Acetone Precipitation and In-solution Digest of C10 Cell Lysate for Phosphoproteomics.** Cultivation of C10 cells is detailed in the main text. Cells were lysed in a buffer comprised of 250 mM MES (pH 6.0), 10mM EDTA, 0.1 mM Neocuproine, 1% SDS, 1% Triton X-100, 75 mM NaCl, 2 µg/ml apoptinin, 10 µg/ml leupeptin, 1 mM phenylmethylsulfonyl fluoride, 10 mM NaF, 1% phosphatase inhibitor cocktail 2 (Sigma-Aldrich), 1% phosphatase inhibitor cocktail 3 (Sigma-Aldrich), and 100 mM NEM. Following lysis and NEM blocking (which are described in the previous sections and the main text), 4x vol. of cold acetone (−20 °C) was added to each tube, which were briefly vortexed and stored at −20 °C overnight. The next day, the samples were centrifuged for 10 min at 13,000 rpm. The supernatant was aspirated, and the pellet was washed twice with 3x vol. of ice-cold acetone. After 2 min drying in air, the pellet was resuspended in 30 µl of 250 mM HEPES (pH 7.0) containing 8 M urea by water bath sonication. Samples were normalized to 200 µg in 30 µL and incubated in 10 mM DTT for 1 hour at 1000 rpm and 37 °C. Samples were then diluted to 120 µL with 50 mM HEPES (pH 7.7). Trypsin was added in a ratio of 1:50, and the samples were incubated overnight at 37 °C with 850 rpm shaking. Subsequent C18 SPE, reduction, and IMAC enrichment steps are detailed in the main text.

References

1. Jumper, J., Evans, R., Pritzel, A., Green, T., Figurnov, M., Ronneberger, O., Tunyasuvunakool, K., Bates, R., Žídek, A., Potapenko, A., Bridgland, A., Meyer, C., Kohl, S. A. A., Ballard, A. J., Cowie, A., Romera-Paredes, B., Nikolov, S., Jain, R., Adler, J., Back, T., Petersen, S., Reiman, D., Clancy, E., Zielinski, M., Steinegger, M., Pacholska, M., Berghammer, T., Bodenstein, S., Silver, D., Vinyals, O., Senior, A. W., Kavukcuoglu, K., Kohli, P., and Hassabis, D. (2021) Highly accurate protein structure prediction with AlphaFold. *Nature* 596, 583–589

2. Leutert, M., Rodríguez‐Mias, R. A., Fukuda, N. K., and Villén, J. (2019) R2‐P2 rapid‐robotic phosphoproteomics enables multidimensional cell signaling studies. *Mol Syst Biol* 15, e9021
